# A generative spike train model with time-structured higher order correlations

**DOI:** 10.3389/fncom.2013.00084

**Published:** 2013-07-17

**Authors:** James Trousdale, Yu Hu, Eric Shea-Brown, Krešimir Josić

**Affiliations:** ^1^Department of Mathematics, University of HoustonHouston, TX, USA; ^2^Department of Applied Mathematics, University of WashingtonSeattle, WA, USA; ^3^Program in Neurobiology and Behavior, University of WashingtonSeattle, WA, USA; ^4^Department of Biology and Biochemistry, University of HoustonHouston, TX, USA

**Keywords:** correlations, spiking neurons, neuronal networks, cumulant, neuronal modeling, neuronal network model, point processes

## Abstract

Emerging technologies are revealing the spiking activity in ever larger neural ensembles. Frequently, this spiking is far from independent, with correlations in the spike times of different cells. Understanding how such correlations impact the dynamics and function of neural ensembles remains an important open problem. Here we describe a new, generative model for correlated spike trains that can exhibit many of the features observed in data. Extending prior work in mathematical finance, this *generalized thinning and shift* (GTaS) model creates marginally Poisson spike trains with diverse temporal correlation structures. We give several examples which highlight the model's flexibility and utility. For instance, we use it to examine how a neural network responds to highly structured patterns of inputs. We then show that the GTaS model is analytically tractable, and derive cumulant densities of all orders in terms of model parameters. The GTaS framework can therefore be an important tool in the experimental and theoretical exploration of neural dynamics.

## 1. Introduction

Recordings across the brain suggest that neural populations spike collectively—the statistics of their activity as a group are distinct from that expected in assembling the spikes from one cell at a time (Bair et al., 2001; Salinas and Sejnowski, 2001; Harris, 2005; Averbeck et al., 2006; Schneidman et al., 2006; Shlens et al., 2006; Pillow et al., 2008; Ganmor et al., 2011; Bathellier et al., 2012; Hansen et al., 2012; Luczak et al., 2013). Advances in electrode and imaging technology allow us to explore the dynamics of neural populations by simultaneously recording the activity of hundreds of cells. This is revealing patterns of collective spiking that extend across multiple cells. The underlying structure is intriguing: For example, higher-order interactions among cell groups have been observed widely (Amari et al., 2003; Schneidman et al., 2006; Shlens et al., 2006, 2009; Ohiorhenuan et al., 2010; Ganmor et al., 2011; Vasquez et al., 2012; Luczak et al., 2013). A number of recent studies point to mechanisms that generate such higher-order correlations from common input processes, including unobserved neurons. This suggests that, in a given recording or given set of neurons projecting downstream, higher-order correlations may be quite ubiquitous (Barreiro et al., 2010; Macke et al., 2011; Yu et al., 2011; Köster et al., 2013). Moreover, these higher-order correlations may impact the firing statistics of downstream neurons (Kuhn et al., 2003), the information capacity of their output (Ganmor et al., 2011; Cain and Shea-Brown, 2013; Montani et al., 2013), and could be essential in learning through spike-time dependent synaptic plasticity (Pfister and Gerstner, 2006; Gjorgjieva et al., 2011).

What exactly is the impact of such collective spiking on the encoding and transmission of information in the brain? This question has been studied extensively, but much remains unknown. Results to date show that the answers will be varied and rich. Patterned spiking can impact responses at the level of single cells (Salinas and Sejnowski, 2001; Kuhn et al., 2003; Xu et al., 2012) and neural populations (Amjad et al., 1997; Tetzlaff et al., 2003; Rosenbaum et al., 2010, 2011). Neurons with even the simplest of non-linearities can be highly sensitive to correlations in their inputs. Moreover, such non-linearities are sufficient to accurately decode signals from the input to correlated neural populations (Shamir and Sompolinsky, 2004).

An essential tool in understanding the impact of collective spiking is the ability to generate artificial spike trains with a predetermined structure across cells and across time (Brette, 2009; Gutnisky and Josić, 2009; Krumin and Shoham, 2009; Macke et al., 2009). Such synthetic spike trains are the grist for testing hypotheses about spatiotemporal patterns in coding and dynamics. In experimental studies, such spike trains can be used to provide structured stimulation of single cells across their dendritic trees via glutamate uncaging (Gasparini and Magee, 2006; Reddy et al., 2008; Branco et al., 2010; Branco and Häusser, 2011). In addition, entire populations of neurons can be activated via optical stimulation of microbial opsins (Han and Boyden, 2007; Chow et al., 2010). Computationally, they are used to examine the response of non-linear models of downstream cells (Carr et al., 1998; Salinas and Sejnowski, 2001; Kuhn et al., 2003).

Therefore, much effort has been devoted to developing statistical models of population activity. A number of flexible, yet tractable probabilistic models of joint neuronal activity have been proposed. Pairwise correlations are the most common type of interactions obtained from multi-unit recordings. Therefore, many earlier models were designed to generate samples of neural activity patterns with predetermined first and second order statistics (Brette, 2009; Gutnisky and Josić, 2009; Krumin and Shoham, 2009; Macke et al., 2009). In these models, higher-order correlations are not explicitly and separately controlled.

A number of different models have been used to analyze higher-order interactions. However, most of these models assume that interactions between different cells are instantaneous (or near-instantaneous) (Kuhn et al., 2003; Johnson and Goodman, 2009; Staude et al., 2010; Shimazaki et al., 2012). A notable exception is the work of Bäuerle and Grübel (2005), which developed such methods for use in financial applications. In these previous efforts, correlations at all orders were characterized by the increase, or decrease, in the probability that groups of cells spike together at the same time, or have a common temporal correlation structure regardless of the group.

The aim of the present work is to provide a statistical method for generating spike trains with more general correlation structures across cells and time. Specifically, we allow distinct temporal structure for correlations at pairwise, triplet, and all higher orders, and do so separately for different groups of cells in the neural population. Our aim to describe a model that can be applied in neuroscience, and can potentially be fit to emerging datasets.

**Figure 1:**
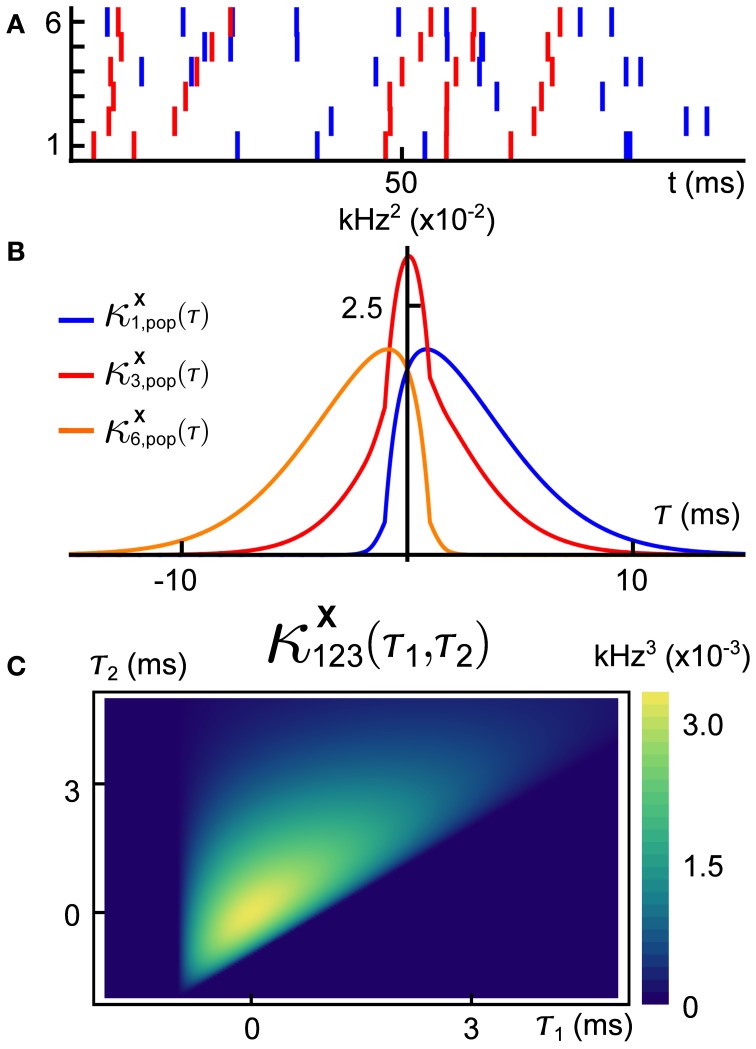
**(A)** Raster plot of event times for an example multivariate Poisson process **X** = (*X*_1_, …, *X*_6_) generated using the methods presented below. This model exhibits independent marginal events (blue) and population-level, chain-like events (red). **(B)** Some second order population cumulant densities (i.e., second order correlation between individual unit activities and population activity) for this model (Luczak et al., 2013). Greater mass to the right (resp. left) of τ = 0 indicates that the cell tends to lead (resp. follow) in pairwise-correlated events. **(C)** Third-order cross-cumulant density for processes *X*_1_, *X*_2_, *X*_3_. The quantity κ^**X**^_123_(τ_1_, τ_2_) yields the probability of observing spikes in cells 2 and 3 at an offset τ_1_, τ_2_ from a spike in cell 1, respectively, in excess of what would be predicted from the first and second order cumulant structure. All quantities are precisely defined in the Methods. Note: system parameters necessary to reproduce results are given in the Appendix for all figures.

A sample realization of a multivariate generalized thinning and shift (GTaS) process is shown in Figure 1. The multivariate spike train consists of six marginally Poisson processes. Each event was either uncorrelated with all other events across the population, or correlated in time with an event in all other spike trains. This model was configured to exhibit activity that cascades through a sequence of neurons. Specifically, neurons with larger index tend to fire later in a population wide event (this is similar to a synfire chain (Abeles, 1991), but with variable timing of spikes within the cascade). In Figure 1B, we plot the “population cross-cumulant density” for three chosen neurons—the summed activity of the population triggered by a spike in a chosen cell. The center of mass of this function measures the average latency by which spikes of the neuron in question precede those of the rest of the population (Luczak et al., 2013). Finally, Figure 1C shows the third-order cross-cumulant density for the three neurons. The triangular support of this function is a reflection of a synfire-like cascade structure of the spiking shown in the raster plot of panel (**A**): when firing events are correlated between trains, they tend to proceed in order of increasing index. We demonstrate the impact of such structured activity on a downstream network in section 2.2.3.

## 2. Results

Our aim is to describe a flexible multivariate point process capable of generating a range of high order correlation structures. To do so, we extend the *TaS* (thinning and shift) model of temporally- and spatially-correlated, marginally Poisson counting processes (Bäuerle and Grübel, 2005). The TaS model itself generalizes the SIP and MIP models (Kuhn et al., 2003) which have been used in theoretical neuroscience (Tetzlaff et al., 2008; Rosenbaum et al., 2010; Cain and Shea-Brown, 2013). However, the TaS model has not been used as widely. The original TaS model is too rigid to generate a number of interesting activity patterns observed in multi-unit recordings (Ikegaya et al., 2004; Luczak et al., 2007, 2013). We therefore developed the *GTaS* which allows for a more diverse temporal correlation structure.

We begin by describing the algorithm for sampling from the GTaS model. This constructive approach provides an intuitive understanding of the model's properties. We then present a pair of examples, the first of which highlights the utility of the GTaS framework. The second example demonstrates how sample point processes from the GTaS model can be used to study population dynamics. Next, we present the analysis which yields the explicit forms for the cross-cumulant densities derived in the context of the examples. We do so by first establishing a useful distributional representation for the GTaS process, paralleling Bäuerle and Grübel (2005). Using this representation, we derive cross-cumulants of a GTaS counting process, as well as explicit expressions for the cross-cumulant densities. After explaining the derivation at lower orders, we present a theorem which describes cross-cumulant densities at all orders.

### 2.1. GTaS model simulation

The GTaS model is parameterized first by a rate λ which determines the intensity of a “mother process”—a Poisson process on ℝ. The events of the mother process are marked, and the markings determine how each event is distributed among a collection of *N* daughter processes. The daughter processes are indexed by the set 𝔻 = {1, …, *N*}, and the set of possible markings is the power set 2^𝔻^, the set of all subsets of 𝔻. We define a probability distribution *p* = (*p*_*D*_)_*D* ⊂ 𝔻_, assigning a probability to each possible marking, *D*. As we will see, *p*_*D*_ determines the probability of a joint event in all daughter processes with indices in the set *D*. Finally, to each marking, *D*, we assign a probability distribution *Q*_*D*_, giving a family of shift (jitter) distributions (*Q*_*D*_)_*D* ⊂ 𝔻_. Each (*Q*_*D*_) is a distribution over ℝ^*N*^.

**Figure 2:**
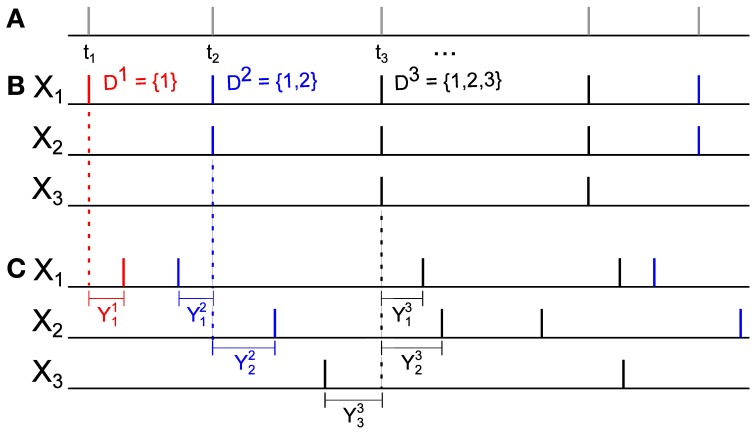
**An illustration of a GTaS simulation. (A)** Step 1: Simulate the mother process—a time-homogeneous Poisson process with event times {t^*j*^}. **(B)** Step 2: For each *t*^*j*^ in step 1, select a set *D*^*j*^ ⊂ 𝔻 according to the distribution *p*_*D*_, and project the event at time *t*^*j*^ to the subsets with indices in *D*^*j*^. The legend indicates the colors assigned to three possible markings in this example. **(C)** Step 3: For each pair (*t*^*j*^, *D*^*j*^) generated in the previous two steps, draw **Y**^*j*^ from *Q*_*D*^*j*^_, and shift the event times in the daughter processes by the corresponding values *Y*^*j*^_*i*_.

The rate λ, the distribution *p* over the markings, and the family of jitter distributions (*Q*_*D*_)_*D* ⊂ 𝔻_, define a vector **X** = (*X*_1_, …, *X*_*N*_) of dependent daughter Poisson processes described by the following algorithm, which yields a single realization (see Figure 2):

1. Simulate the mother Poisson process of rate λ on ℝ, generating a sequence of event times {*t*^*j*^}. (Figure 2A).
2. With probability *p*_*D*^*j*^_ assign the subset *D*^*j*^ ⊂ 𝔻 to the event of the mother process at time *t*^*j*^. This event will be assigned only to processes with indices in *D*^*j*^. (Figure 2B).
3. Sample a vector (*Y*^*j*^_1_, …, *Y*^*j*^_*N*_) = **Y**^*j*^ from the distribution *Q*_*D*^*j*^_. For each *i* ∈ *D*, the time *t*^*j*^ + *Y*^*j*^_*i*_ is set as an event time for the marginal counting process *X*_*i*_. (Figure 2C).

Hence copies of each point of the mother process are placed into daughter processes after a shift in time. A primary difference between the GTaS model and the TaS model presented in Bäuerle and Grübel (2005) is the dependence of the shift distributions *Q*_*D*_ on the chosen marking. This allows for greater flexibility in setting the temporal cumulant structure.

### 2.2. Examples

#### 2.2.1. Relation to SIP/MIP processes

Two simple models of correlated, jointly Poisson processes were defined in Kuhn et al. (2003). The resulting spike trains exhibit spatial correlations, but only instantaneous temporal dependencies. Each model was constructed by starting with independent Poisson processes, and applying one of two elementary point process operations: superposition and thinning (Cox and Isham, 1980). We show that both models are special cases of the GTaS model.

In the *single interaction process* (SIP), each marginal process *X*_*i*_ is obtained by merging an independent Poisson process with a common, global Poisson process. That is,

$$X_{i}(\cdot )=Z_{i}(\cdot )+Z_{c}(\cdot ),\;i=1,\ldots ,N,$$

where *Z*_*c*_ and each *Z*_*i*_ are independent Poisson counting processes on ℝ with rates λ_*c*_,λ_*i*_, respectively. An SIP model is equivalent to a GTaS model with mother process rate λ = λ_*c*_ + ∑^*N*^_*i* = 1_ λ_*i*_, and marking probabilities

$$p_{D}=\{\begin{array}{ll} \frac{\lambda_{i}}{\lambda } & D=\{i\}\\ \frac{\lambda_{c}}{\lambda } & D=𝔻\\ 0 & \text{otherwise} \end{array}.$$

Note that if λ_*c*_ = 0, each spike will be assigned to a different process *X*_*i*_, resulting in *N* independent Poisson processes. Lastly, each shift distribution is equal to a delta distribution at zero in every coordinate (i.e., *q*_*D*_(*y*_1_, …, *y*_*N*_) ≡ ∏^*N*^_*i* = 1_δ(*y*_*i*_) for every *D* ⊂ 𝔻). Thus, all joint cumulants (among distinct marginal processes) of orders 2 through *N* are delta functions of equal magnitude, λ*p*_𝔻_.

The *multiple interaction process* (MIP) consists of *N* Poisson processes obtained from a common mother process with rate λ_*m*_ by *thinning* (Cox and Isham, 1980). The *i*th daughter process is formed by independent (across coordinates and events) deletion of events from the mother process with probability *p* = (1 − ϵ). Hence, an event is common to *k* daughter processes with probability ϵ^*k*^. Therefore, if we take the perspective of retaining, rather than deleting events, the MIP model is equivalent to a GTaS process with λ = λ_*m*_, and *p*_*D*_ = ϵ^|*D*|^(1 − ϵ)^*d* − |*D*|^. As in the SIP case, the shift distributions are singular in every coordinate. Below, we present a general result (Theorem 1.1) which immediately yields as a corollary that the MIP model has cross-cumulant functions which are δ functions in all dimensions, scaled by ϵ^*k*^, where *k* is the order of the cross-cumulant.

#### 2.2.2. Generation of synfire-like cascade activity

The GTaS framework provides a simple, tractable way of generating cascading activity where cells fire in a preferred order across the population—as in a synfire chain, but (in general) with variable timing of spikes (Abeles, 1991; Abeles and Prut, 1996; Aertsen et al., 1996; Aviel et al., 2002; Ikegaya et al., 2004). More generally, it can be used to simulate the activity of *cell assemblies* (Hebb, 1949; Harris, 2005; Buzsáki, 2010; Bathellier et al., 2012), in which the firing of groups of neurons is likely to follow a particular order.

**Figure 3:**
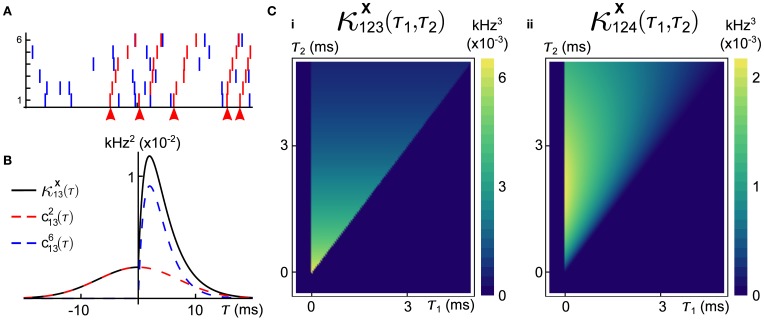
**An example of a six dimensional GTaS model exhibiting synfire-like cascading firing patterns. (A)** A raster-plot of spiking activity over a 100 ms window. Blue spikes indicate either marginal or pairwise events (i.e., events corresponding to markings for sets *D* ⊂𝔻 with |*D*| ≤ 2). Red spikes indicate population-wide events which have shift-times given by cumulative sums of independent exponentials, as described in the text. Arrows indicate the location of the first spike in the cascade. **(B)** A second-order cross-cumulant κ^**X**^_13_ (black line) of this model is composed of contributions from two sources: correlations due to second-order markings, which have Gaussian shifts (*c*^2^_13_—dashed red line), and correlations due to the occurrence of population wide events (*c*^*N*^_13_—dashed blue line). **(C)** Density plots of the third-order cross-cumulant density for triplets **(i)** (1, 2, 3) and **(ii)** (1, 2, 4)—the latter is given explicitly in Equation (6). System parameters are given in the Appendix.

In the Introduction, we briefly presented one example in which the GTaS framework was used to generate synfire-like cascade activity (see Figure 1), and we present another in Figure 3. In what follows, we will present the explicit definition of this second model, and then derive explicit expressions for its cumulant structure. Our aim is to illustrate the diverse range of possible correlation structures that can be generated using the GTaS model.

Consider an *N*-dimensional counting process **X** = (*X*_1_, …, *X*_*N*_) of GTaS type, where *N* ≥ 4. We restrict the marking distribution so that *p*_*D*_ ≡ 0 unless |*D*| ≤ 2 or *D* = 𝔻. That is, events are either assigned to a single, a pair, or all daughter processes. For sets *D* with |*D*| = 2, we set 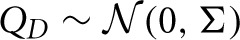
—a Gaussian distributions of zero mean and some specified covariance. The choice of the precise pairwise shift distributions is not important. Shifts of events attributed to a single process have no effect on the statistics of the multivariate process—this will become clear in section 2.3, when we exhibit that a GTaS process is equivalent in distribution to a sum of independent Poisson processes. In that context, the shifts of marginal events are applied to the event times of only one of these Poisson processes, which does not impact its statistics.

It remains to define the jitter distribution for events common to the entire population of daughter processes, i.e., events marked by 𝔻. We will show that we can generate cascading activity, and analytically describe the resulting correlation structure. We will say that a random variable *T* follows the exponential distribution Exp(α) if it has probability density

$$f(t|\alpha )=\alpha e^{-\alpha t}\Theta (t),$$

where Θ(*t*) is the Heaviside step function. We generate random vectors **Y** ~ *Q*_𝔻_ according to the following rule, for each *i* = 1, …, *N*:

1. Generate independent random variables *T*_*i*_ ~ Exp(α_*i*_) where α_*i*_ > 0.
2. Set *Y*_*i*_ = ∑^*i*^_*j* = 1_ *T*_*j*_.

In particular, note that these shift times satisfy *Y*_*N*_ ≥… ≥ *Y*_2_ ≥ *Y*_1_ ≥ 0, indicating the chain-like structure of these joint events.

From the definition of the model and our general result (Theorem 1.1) below, we immediately have that κ^**X**^_*ij*_(τ), the second order cross-cumulant density for the process (*i, j*), is given by

(1) $$\kappa_{ij}^{X}(\tau )=c_{ij}^{2}(\tau )+c_{ij}^{N}(\tau ),$$

where

(2) $$\begin{array}{l} c_{ij}^{2}(\tau )=\lambda p_{\left\{i,j\right\}}\int q_{\left\{i,j\right\}}^{\left\{i,j\right\}}(t,t+\tau )dt,\\ c_{ij}^{N}(\tau )=\lambda p_{𝔻}\int q_{𝔻}^{\left\{i,j\right\}}(t,t+\tau )dt \end{array}$$

define the contributions to the second order cross-cumulant density by the second-order, Gaussian-jittered events and the population-level events, respectively. Therefore, correlations between spike trains in this case reflect distinct contributions from second order and higher order events. The functions *q*^*D*′^_*D*_ indicate the densities associated with the distribution *Q*_*D*_, projected to the dimensions of *D*′. All statistical quantities are precisely defined in the methods.

By exploiting the hierarchical construction of the shift times, we can find an expression for the joint density *q*_𝔻_, necessary to explicitly evaluate Equation (1). For a general *N*-dimensional distribution,

(3) $$f(y_{1},\ldots ,y_{N})=f(y_{N}|y_{1},\ldots ,y_{N-1})f(y_{N-1}|y_{1},\ldots ,y_{N-2})\cdots \cdot f(y_{2}|y_{1})f(y_{1}).$$

Since *Y*_1_ ~ Exp(α_1_), we have *f*(*y*_1_) = exp[− α_1_*y*_1_]Θ(*y*_1_), where Θ(*y*) is the Heaviside step function. Further, as (*Y*_*i*_ − *Y*_*i* − 1_) | (*Y*_1_, …, *Y*_*i* − 1_) ~ Exp(α_*i*_) for *i* ≥ 2, the conditional densities of the *y*_*i*_'s take the form

$$\begin{array}{l} f(y_{i}|y_{1},\ldots ,y_{i-1})=f(y_{i}|y_{i-1})=\alpha_{i}\exp\left[-\alpha_{i}(y_{i}-y_{i-1})\right]\\ \;\cdot \Theta \;(y_{i}-y_{i-1}),\;i\geq 2. \end{array}$$

Substituting this in to the identity Equation (3), we have

(4) $$q_{𝔻}(y_{1},\ldots ,y_{N})=\{\begin{array}{cc} \alpha_{1}\exp\left[-\alpha_{1}y_{1}\right]\prod_{i=2}^{N}\alpha_{i} & y_{N}\geq \ldots \geq \\ \;\cdot \exp\left[-\alpha_{i}(y_{i}-y_{i-1})\right] & \;y_{2}\geq y_{1}\geq 0\\ 0 & \text{otherwise} \end{array}.$$

Using Theorem 1.1 (Equation A8) we obtain the *N*th order cross-cumulant density (see the Methods),

(5) $$\begin{array}{l} \kappa_{1\cdots N}^{X}(\tau_{1},\ldots ,\tau_{N-1})\\ =\lambda p_{𝔻}\int q_{𝔻}(t,t+\tau_{1},\ldots ,t+\tau_{N-1})dt\\ =\lambda p_{𝔻}\cdot \{\begin{array}{cc} \prod_{i=1}^{N-1}\alpha_{i+1} & \tau_{i}\geq \tau_{i-1}\\ \;\cdot \exp\left[-\alpha_{i+1}(\tau_{i}-\tau_{i-1})\right] & \;i=1,\ldots ,N-1,\\ 0 & \text{otherwise} \end{array} \end{array}$$

where, for notational convenience, we define τ_0_ = 0. A raster plot of a realization of this model is shown in Figure 3A. We note that the cross-cumulant densities of arbitrary subcollections of the counting processes **X** can be obtained by finding the appropriate marginalization of *q*_𝔻_ via integration of Equation (4). In the case that common distributions are used to define the shifts, symbolic calculation environments (i.e., Mathematica) can quickly yield explicit formulas for cross-cumulant densities. Mathematica notebooks for Figure 1 available upon request.

As a particular example, we consider the cross-cumulant density of the marginal processes *X*_1_, *X*_3_. Using Equations (2, 4), we find

$$c_{13}^{N}(\tau )=\lambda p_{𝔻}\Theta (\tau )\cdot \{\begin{array}{ll} \frac{\alpha_{2}\alpha_{3}}{\alpha_{3}-\alpha_{2}}\left\{\exp\left[-\alpha_{2}\tau \right]-\exp\left[-\alpha_{3}\tau \right]\right\} & \alpha_{2}\neq \alpha_{3}\\ \alpha_{2}\alpha_{3}\tau \exp\left[-\alpha_{2}\tau \right] & \alpha_{2}=\alpha_{3} \end{array}.$$

An expression for *c*^2^_13_(τ) may be obtained similarly using Equation (2) and recalling that 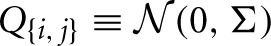 for all *i, j*. In Figure 3B, we plot these contributions, as well as the full covariance density.

Similar calculations at third order yield, as an example,

(6) $$\begin{array}{l} \kappa_{124}^{X}(\tau_{1},\tau_{2})=\lambda p_{𝔻}\\ \cdot \{\begin{array}{ll} \frac{\alpha_{2}\alpha_{3}\alpha_{4}}{\alpha_{4}-\alpha_{3}}\exp\left[-\alpha_{2}\tau_{1}\right]\{\exp\left[-\alpha_{3}(\tau_{2}-\tau_{1})\right] & \\ \;-\exp\left[-\alpha_{4}(\tau_{2}-\tau_{1})\right]\} & \alpha_{3}\neq \alpha_{4}\\ \alpha_{2}\alpha_{3}\alpha_{4}(\tau_{2}-\tau_{1})\exp\left[-\alpha_{2}\tau_{1}-\alpha_{3}(\tau_{2}-\tau_{1})\right] & \alpha_{3}=\alpha_{4} \end{array}\text{​​}, \end{array}$$

where the cross-cumulant density κ^**X**^_124_(τ_1_,τ_2_) is supported only on τ_2_ ≥ τ_1_ ≥ 0. Plots of the third-order cross-cumulants for triplets (1, 2, 3) and (1, 2, 4) in this model are shown in Figure 3C. Note that, for the specified parameters, the conditional distribution of *Y*_4_—the shift applied to the events of *X*_4_ in a joint population event—given *Y*_2_ follows a gamma distribution, whereas *Y*_3_ | *Y*_2_ follows an exponential distribution, explaining the differences in the shapes of these two cross-cumulant densities.

General cross-cumulant densities of at least third order for the cascading model will have a form similar to that given in Equation (6), and will contain no signature of the correlation of strictly second order events. This highlights a key benefit of cumulants as a measure of dependence: although they agree with central moments up to third order, we know from Equation (23) below [or Equation (22) in the general case] that central moments necessarily exhibit a dependence on lower order statistics. On the other hand, cumulants are “pure” and quantify only dependencies at the given order which cannot be inferred from lower order statistics (Grün and Rotter, 2010).

One useful statistic for analyzing population activity through correlations is the *population cumulant density* (Luczak et al., 2013). The second order population cumulant density for cell *i* is defined by (see the Methods)

$$\kappa_{i,\text{pop}}^{X}(\tau )=\sum_{j\neq i}\kappa_{ij}^{X}(\tau ).$$

**Figure 4:**
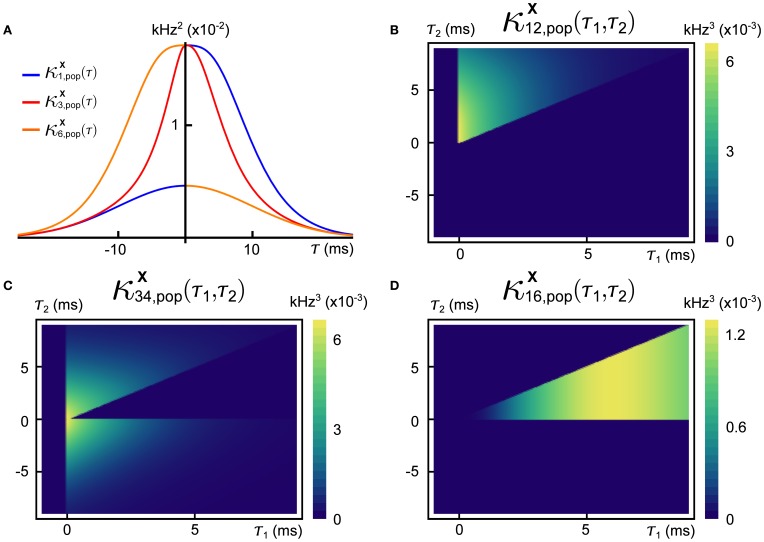
**Population cumulants for the synfire-like cascading GTaS process of Figure 3.** See Equation (25) for the definition of population cumulants. **(A)** Second order population cumulant densities for processes 1, 3, and 6. Greater mass to the right (resp. left) of τ = 0 indicates that a cell tends to lead (resp. follow) in pairwise-correlated events. **(B)** Third order population cumulant for processes *X*_1_, *X*_2_ in the cascading GTaS process. Concentration of the mass in different regions of the plane indicates temporal structure of events correlated between *X*_1_, *X*_2_ relative to the remainder of the population (see the text). **(C)** Same as **(B)**, but for processes *X*_3_, *X*_4_. **(D)** Same as **(B)**, but for processes *X*_1_, *X*_6_. System parameters are given in the Appendix.

This function is linearly related to the spike-triggered average of the population activity conditioned on that of cell *i*. In Figure 4 we show three different second-order population-cumulant functions for the cascading GTaS model of Figure 3A. When the second order population cumulant for a neuron is skewed to the right of τ = 0 (as is κ^**X**^_1, pop_—blue line), a neuron tends to precede other cells in the population in pairwise spiking events. Similarly, skewness to the left of τ = 0 (κ^**X**^_6, pop_—orange line) indicates a neuron which tends to trail other cells in the population in such events. A symmetric population cumulant density indicates a neuron is a follower *and* a leader. Taken together, these three second order population cumulants hint at the chain structure of the process.

Greater understanding of the joint temporal statistics in a multivariate counting process can be obtained by considering higher-order population cumulant densities. We define the third-order population cumulant density for the pair (*i, j*) to be

$$\kappa_{ij,\text{pop}}^{X}(\tau_{1},\tau_{2})=\sum_{k\neq i,j}\kappa_{ijk}^{X}(\tau_{1},\tau_{2}).$$

The third-order population cumulant density is linearly related to the spike-triggered population activity, conditioned on spikes in cells *i* and *j* separated by a delay τ_1_. In Figures 4B–D, we present three distinct third-order population cumulant densities. Examining κ^**X**^_12, pop_(τ_1_, τ_2_) (panel **B**), we see only contributions in the region τ_2_ > τ_1_ > 0, indicating that the pairwise event 1 → 2 often precedes a third spike elsewhere in the population (i.e., with a probability above chance). The population cumulant κ^**X**^_34, pop_(τ_1_, τ_2_) has contributions in two sections of the plane (panel **C**). Contributions in the region τ_2_ > τ_1_ > 0 can be understood following the preceding example, while contributions in the region τ_2_ < 0 < τ_1_ imply that the firing of other neurons tends to precede the joint firing event 3 → 4. Lastly, contributions to κ^**X**^_16, pop_(τ_1_, τ_2_) (panel **D**) are limited to 0 < τ_2_ < τ_1_, indicating an above chance probability of joint firing events of the form 1→ *i* → 6, where *i* indicates a distinct neuron within the population.

A distinct advantage of the study of population cumulant densities as opposed to individual cross-cumulant functions in practical applications is related to data (i.e., sample size) limitations. In many practical applications, where the temporal structure of a collection of observed point processes is of interest, we often deal with a small, noisy samples. It may therefore be difficult to estimate third- or higher-order cumulants. Population cumulants partially circumvent this issue by *pooling* (Tetzlaff et al., 2003; Rosenbaum et al., 2010, 2011) (or summing) responses, to amplify existing correlations and average out the noise in measurements.

We conclude this section by noting that even cascading GTaS examples can be much more general. For instance, we can include more complex shift patterns, overlapping subassemblies within the population, different temporal processions of the cascade, and more.

#### 2.2.3. Timing-selective network

The responses of single neurons and neuronal networks in experimental (Meister and Berry II, 1999; Singer, 1999; Bathellier et al., 2012) and theoretical studies (Jeffress, 1948; Hopfield, 1995; Joris et al., 1998; Thorpe et al., 2001; Gütig and Sompolinsky, 2006) can reflect the temporal structure of their inputs. Here, we present a simple example that shows how a network can be selective to fine temporal features of its input, and how the GTaS model can be used to explore such examples.

As a general network model, we consider *N* leaky integrate-and-fire (LIF) neurons with membrane potentials *V*_*i*_ obeying

(7) $$\frac{dV_{i}}{dt}=-V_{i}+\sum_{j=1}^{N}w_{ij}(F*z_{j})(t)+w^{\text{in}}x_{i}(t),\;i=1,\ldots ,N.$$

When the membrane potential of cell *i* reaches a threshold *V*th, an output spike is recorded and the membrane potential is reset to zero, after which evolution of *V*_*i*_ resumes the dynamics in Equation (7). Here *w*_*ij*_ is the synaptic weight of the connection from cell *j* to *i*, *w*^in^ is the input weight, and we assume time to be measured in units of membrane time constants. The function *F* = τ^− 1^_syn_ *e*^− (*t* − τ_d_)/τ_syn_^Θ(*t* − τ_d_) is a delayed, unit-area exponential synaptic kernel with time-constant τ_syn_ and delay τ_d_. The output of the *i*th neuron is

$$z_{i}(t)=\sum_{j}\delta (t-t_{i}^{j}),$$

where *t*^*j*^_*i*_ is the time of the *j*th spike of neuron *i*. In addition, the input {*x*_*i*_}^*N*^_*i* = 1_ is

$$x_{i}(t)=\sum_{j}\delta (t-s_{i}^{j}),$$

where the event times {*s*^*j*^_*i*_} correspond to those of a GTaS counting process **X**. Thus, each input spike results in a jump in the membrane potential of the corresponding LIF neuron of amplitude *w*^in^. The particular network we consider will have a ring topology (nearest neighbor-only connectivity)—specifically, for *i, j* = 1, …, *N*, we let

$$w_{ij}=\{\begin{array}{ll} w^{\text{syn}} & i-j\;\mathrm{mod}\;N\equiv 1\text{ or }N-1\\ 0 & \text{otherwise} \end{array}.$$

We further assume that all neurons are *excitatory*, so that *w*^syn^ > 0.

A network of LIF neurons with synaptic delay is a minimal model which can exhibit fine-scale discrimination of temporal patterns of inputs without precise tuning (Izhikevich, 2006) (that is, without being carefully designed to do so, with great sensitivity to modification of network parameters). To exhibit this dependence we generate inputs from two GTaS processes. The first (the *cascading model*) was described in the preceding example. To independently control the mean and variance of relative shifts we replace the sum of exponential shifts with sums of gamma variates. We also consider a model featuring population-level events without shifts (the *synchronous model*), where the distribution *Q*_𝔻_ is a δ distribution at zero in all coordinates.

The only difference between the two input models is in the temporal structure of joint events. In particular, the rates, and all long timescale spike count cross-cumulants (equivalent to the total “area” under the cross-cumulant density, see the Methods) of order two and higher are identical for the two processes. We focus on the sensitivity of the network to the temporal cumulant structure of its inputs.

**Figure 5:**
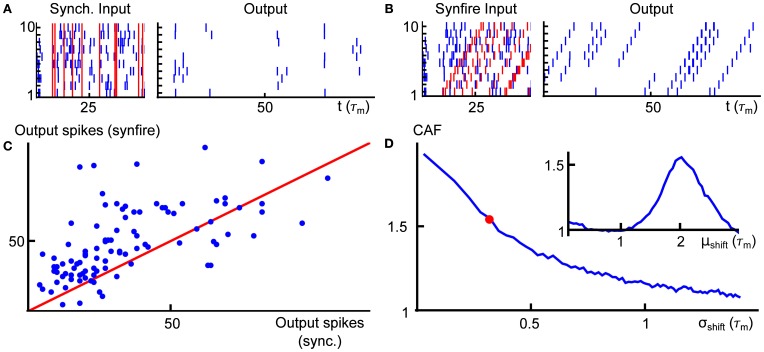
**(A)** Example input (left) and output (right) for the nearest neighbor LIF network receiving input with synchronous input. **(B)** Same as **(A)**, but for cascading input. **(C)** Scatter plot of the output spike count of the network receiving synchronous (horizontal axis) and cascading input (vertical axis) with μ_shift_ = 2, σ_shift_ = 0.3. The red line is the diagonal. **(D)** Average gain (rate in response to cascading input divided by rate in response to synchronous input) as a function of the standard deviation of the gamma variates which compose the shift vectors for population-level events (μ_shift_ was fixed at 2). The red dot indicates the value of σ_shift_ used in panel **(C)**. Inset shows the same gain as panel **(D)**, but for varying the mean of the shift distribution (σ_shift_ = 0.3). Spike counts in panels **(C,D)** were obtained for trials of length *T* = 100. Other system parameters are given in the Appendix.

In Figures 5A,B, we present two example rasters of the nearest-neighbor LIF network receiving synchronous (left) and cascading (right) input. In the second case, there is an obvious pattern in the outputs, but the firing rate is also increased. This is quantified in Figure 5C, where we compare the number of output spikes fired by a network receiving synchronous input (horizontal axis) with the same for a network receiving cascading input (vertical axis), over a large number of trials. On average, the cascading input increases the output rate by a factor of 1.5 over the synchronous inputs—we refer to this quantity as the *cascade amplification factor* (CAF).

Finally, in Figure 5D, we illustrate how the cascade amplification factor depends on the parameters that define the timing of spikes for the cascading inputs. First, we study the dependence on the standard deviation σ_shift_ of the gamma variates determining the shift distribution. We note that amplification factors above 1.5 hold robustly (i.e., for a range of shift σ_shift_ values). The amplification factors decrease with shift variance. In the inset to panel (**D**), we show how the gain depends on the mean of the shift distribution μ_shift_. On an individual trial, the response intensity will depend strongly on the total number of input spikes. Thus, in order to enforce a fair comparison, the mother process and markings used were identical in each trial of every panel of Figure 5. We note that network properties, such as the membrane properties of individual cells or synaptic timescales, may have an equally large impact on the cascade amplification factor—indeed, as we explain below, the observed behavior of the CAF is a result of synergy between the timescales of input and interactions within the network.

These observations have simple explanations in terms of the network dynamics and input statistics. Neglecting, for a moment, population-level events, the network is configured so that correlations in activity decrease with topographic distance. Accordingly, the probability of finding neurons that are simultaneously close to threshold also decreases with distance. Under the synchronous input model, a population-level event results in a simultaneous increase of the membrane potentials of all neurons by an amount *w*^in^, but unless the input is very strong (in which case every, or almost every, neuron will fire regardless of fine-scale input structure), the set of neurons sufficiently close to threshold to “capitalize” on the input and fire will typically be restricted to a topographically adjacent subset. Neurons which do not fire almost immediately will soon have forgotten about this population-level input. As a result, the output does not significantly reflect the chain-like structure of the inputs (Figure 5A, right).

On the other hand, in the case of the cascading input, the temporal structure of the input and the timescale of synapses can operate synergistically. Consider a pair of adjacent neurons in the ring network, called cells 1 and 2, arranged so that cell 2 is downstream from cell 1 in the direction of the population-level chain events. When cell 1 spikes, it is likely that cell 2 will also have an elevated membrane potential. The potential is further elevated by the delayed synaptic input from cell 1. If cell 1 spikes in response to a population-level chain event, then cell 2 imminently receives an input spike as well. If the synaptic filter and time-shift of the input spikes to each cell align, then the firing probability of cell 2 will be large relative to chance. This reasoning can be carried on across the network. Hence synergy between the temporal structure of inputs and network architecture allows the network to selectively respond to the temporal structure of the inputs (Figure 5B, right).

In Kuhn et al. (2003), the effect of higher order correlations on the firing rate gain of an integrate-and-fire neuron was studied by driving single cells using sums of SIP or MIP processes with equivalent firing rates (first order cumulants) and pairwise correlations (second order cumulants). In contrast, in the preceding example, the two inputs have equal long time spike count cumulants, and differ only in temporal correlation structure. An increase in firing rate was due to network interactions, and is therefore a population level effect. We return to this comparison in the Discussion.

These examples demonstrate how the GTaS model can be used to explore the impact of spatio-temporal structure in population activity on network dynamics. We next proceed with a formal derivation of the cumulant structure for a general GTaS process.

### 2.3. Cumulant structure of a GTaS process

The GTaS model defines an *N*-dimensional counting process. Following the standard description for a counting process, **X** = (*X*_1_, …, *X*_*N*_) on ℝ^*N*^, given a collection of Borel subsets 
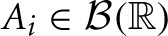
, *i* = 1, …, *N*, then **X**(*A*_1_ × … × *A*_*N*_) = (*X*_1_(*A*_1_), …, *X*_*N*_(*A*_*N*_)) ∈ ℕ^*N*^ is a random vector where the value of each coordinate *i* indicates the (random) number of points which fall inside the set *A*_*i*_. Note that the GTaS model defines processes that are marginally Poisson. All GTaS model parameters and related quantities are defined in Table 1.

**Table 1:** **Common notation used in the text**.

𝔻	𝔻 = {1, 2, …, *N*} where *N* is the system size of the GTaS process under consideration
(*p*_*D*_)_*D* ⊂ 𝔻_	Marking probabilities of a GTaS process
(*Q*_*D*_)_*D* ⊂ 𝔻_	Family of shift distributions on ℝ^*N*^ for a GTaS process
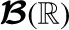	Borel subsets of the real line ℝ
ξ(*D*; *A*_1_, …, *A*_*N*_)	Independent Poisson variables which count points which, after shifting, lie in the sets *A*_*i*_ only along the dimensions corresponding to the indices of *D*. These counts consist of contributions from subsets marked for *D*′ ⊃ *D*, but indices in *D*′\*D* end up outside the corresponding *A*_*i*_. Defined in the statement of Theorem 0
ζ_*D*_(*A*_1_, …, *A*_*N*_)	Independent Poisson variables which are context-dependent resummations of the variables ξ(*D*; *A*_1_, …, *A*_*N*_). Defined below Equation (10)
κ(*X*_1_, …, *X*_*N*_)	Cross-cumulant of the random variables *X*_1_, …, *X*_*N*_ defined in the Methods
κ^**X**^_*i*_1_ … *i*_*k*__(τ_1_, …, τ_*k* − 1_)	Cross-cumulant density defined in Equation (24)
κ^**X**^_*i*_1_ … *i*_*k* − 1_, pop_(τ_1_, …, τ_*k* − 1_)	Population cumulant density defined in Equation (25)

For each *D* ⊂ 𝔻 = {1, …, *N*}, define the tail probability ${\bar{p}}_{D}$ by

(8) $${\bar{p}}_{D}=\sum_{D\subset D^{\prime }\subset 𝔻}p_{D^{\prime }}.$$

Since *p*_*D*_ is the probability that exactly the processes in *D* are marked, ${\bar{p}}_{D}$ is the probability that all processes in *D*, as well as possibly other processes, are marked. An event from the mother process is assigned to daughter process *X*_*i*_ with probability ${\bar{p}}_{\left\{i\right\}}$. As noted above, an event attributed to process *i* following a marking *D* ∋ *i* will be marginally shifted by a random amount determined by the distribution *Q*^{*i*}^_*D*_ which represents the projection of *Q*_*D*_ onto dimension *i*. Thus, the events in the marginal process *X*_*i*_ are shifted in an independent and identically distributed (IID) manner according to the mixture distribution *Q*_*i*_ given by

$$Q_{i}=\frac{\sum_{D\;∋\;i}p_{D}Q_{D}^{\left\{i\right\}}}{\sum_{D\;∋\;i}p_{D}}.$$

Note that IID shifting of the event times of a Poisson process generates another Poisson process of identical rate. Thus, the process *X*_*i*_ is marginally Poisson with rate $\lambda {\bar{p}}_{\left\{i\right\}}$ (Ross, 1995).

In deriving the statistics of the GTaS counting process **X**, it will be useful to express the distribution of **X** as

(9) $$\left(\begin{array}{c} X_{1}(A_{1})\\ \vdots \\ X_{N}(A_{N}) \end{array}\right){=}_{\text{distr}}\left(\begin{array}{c} \sum_{D∋1}\xi (D;A_{1},\ldots ,A_{N})\\ \vdots \\ \sum_{D∋N}\xi (D;A_{1},\ldots ,A_{N}) \end{array}\right).$$

Here, each ξ(*D*; *A*_1_, …, *A*_*N*_) is an independent Poisson process, and the notation =_distr_ indicates that the two random vectors are equal in distribution. This process counts the number of points which are marked by a set *D*′ ⊃ *D*, but (after shifting) only the points with indices *i* ∈ *D* lie in the corresponding set *A*_*i*_. Precise definitions of the processes ξ and a proof of Equation (9) may be found in the Appendix. We emphasize that the Poisson processes ξ(*D*) do not directly count points marked for the set *D*, but instead points which are marked for a set containing *D* that, after shifting, only have their *D*-components lying in the “relevant” sets *A*_*i*_.

Suppose we are interested in calculating dependencies among a subset of daughter processes, ${\left\{X_{i_{j}}\right\}}_{i_{j}\in \bar{D}}$ for some set $\bar{D}\subset 𝔻$, consisting of $|\bar{D}|=k$ distinct members of the collection of counting processes **X**. Then the following alternative representation will be useful:

(10) $$\left(\begin{array}{c} X_{i_{1}}(A_{i_{1}})\\ \vdots \\ X_{i_{k}}(A_{i_{k}}) \end{array}\right){=}_{\text{distr}}\left(\begin{array}{c} \sum_{i_{1}\in D\subset \bar{D}}\zeta_{D}(A_{1},\ldots ,A_{N})\\ \vdots \\ \sum_{i_{k}\in D\subset \bar{D}}\zeta_{D}(A_{1},\ldots ,A_{N}) \end{array}\right)$$

where

$$\zeta_{D}(A_{1},\ldots ,A_{N})=\sum_{\begin{array}{l} \;D^{\prime }⊃D\\ (\bar{D}\backslash D)\cap D^{\prime }=\emptyset \end{array}}\xi (D^{\prime };A_{1},\ldots ,A_{N}).$$

**Figure 6:**
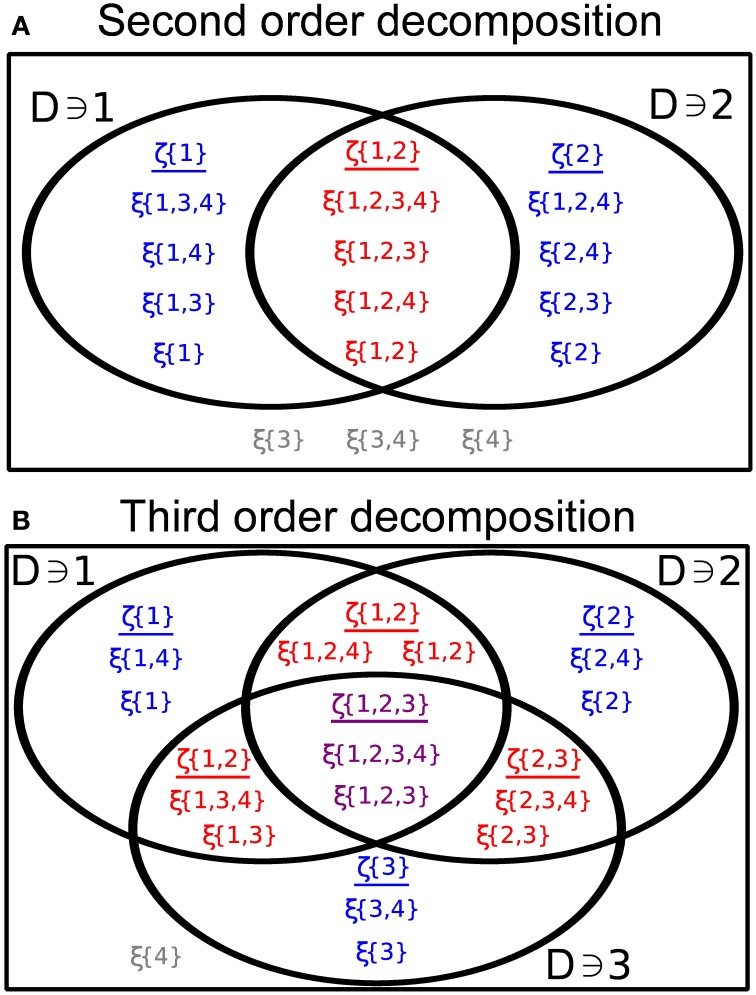
**(A)** Illustrating the representation given by Equation (10) in the case of two distinct processes (see Equation 11) with *N* = 4 and $\bar{D}=\{1,2\}$. **(B)** Same as **(A)**, for three processes with $\bar{D}=\{1,2,3\}$ (see Equation 16).

We illustrate this decomposition in the cases *k* = 2, 3 in Figure 6. The sums in Equation (10) run over all sets *D* ⊂ 𝔻 containing the indicated indices *i*_*j*_ and contained within $\bar{D}$. The processes ζ_*D*_ are comprised of a sum of all of the processes ξ(*D*′) (defined below Equation 9) such that *D*′ contains all of the indices *D*, but no other indices which are part of the subset $\bar{D}$ under consideration. These sums are non-overlapping, implying that the ζ_*D*_ are also independent and Poisson.

The following examples elucidate the meaning and significance of Equation (10). We emphasize that the GTaS process is a completely characterized, joint Poisson process, and we use Equation (10) to calculate cumulants of a GTaS process. In principle, any other statistics can be obtained similarly.

#### 2.3.1. Second order cumulants (covariance)

We first generalize a well-known result about the dependence structure of temporally jittered pairs of Poisson processes, *X*_1_, *X*_2_. Assume that events from a mother process with rate λ, are assigned to two daughter processes with probability *p*. Each event time is subsequently shifted independently according to a univariate distribution *f*. The cross-cumulant density (or cross-covariance function; see the Methods for cumulant definitions) then has the form (Brette, 2009)

$$\kappa_{12}^{X}(\tau )=\lambda p\int f(t)f(t+\tau )dt=\lambda p(f\times f)(\tau ).$$

We generalize this result within the GTaS framework. At second order, Equation (10) has a particularly nice form. Following Bäuerle and Grübel (2005) we write for *i* ≠ *j* (see Figure 6A)

(11) $$\left(\begin{array}{c} X_{i}(A_{i})\\ X_{j}(A_{j}) \end{array}\right){=}_{\text{distr}}\left(\begin{array}{c} \zeta_{\left\{i,j\right\}}(A_{i},A_{j})+\zeta_{\left\{i\right\}}(A_{i})\\ \zeta_{\left\{i,j\right\}}(A_{i},A_{j})+\zeta_{\left\{j\right\}}(A_{j}) \end{array}\right).$$

The process ζ_{*i, j*}_ sums all ξ(*D*′) for which {1, 2} ⊂ *D*′, while the process ζ_{*i*}_ sums all ξ(*D*′) such that *i* ∈ *D*′, *j* ∉ *D*′, and ζ_{*j*}_ is defined likewise.

Using the representation in Equation (11), we can derive the second order cumulant (covariance) structure of a GTaS process. First, we have

$$\begin{array}{l} \mathrm{cov}[X_{i}(A_{i}),X_{j}(A_{j})]=\kappa [X_{i}(A_{i}),X_{j}(A_{j})]\\ \;=\kappa [\zeta_{\left\{i,j\right\}}(A_{i},A_{j}),\zeta_{\left\{i,j\right\}}(A_{i},A_{j})]\\ \;+\kappa [\zeta_{\left\{i\right\}}(A_{i}),\zeta_{\left\{i,j\right\}}(A_{i},A_{j})]\\ \;+\kappa [\zeta_{\left\{i,j\right\}}(A_{i},A_{j}),\zeta_{\left\{j\right\}}(A_{j})]\\ \;+\kappa [\zeta_{\left\{i\right\}}(A_{i}),\zeta_{\left\{j\right\}}(A_{j})]\\ \;=\kappa_{2}[\zeta_{\left\{i,j\right\}}(A_{i},A_{j})]+0\\ \;=E[\zeta_{\left\{i,j\right\}}(A_{i},A_{j})]. \end{array}$$

The third equality follows from the construction of the processes ζ_*D*_: if *D* ≠ *D*′, then the processes ζ_*D*_, ζ_*D*′_ are independent. The final equality follows from the observation that every cumulant of a Poisson random variable equals its mean.

The covariance may be further expressed in terms of model parameters (see Theorem 1.1 for a generalization of this result to arbitrary cumulant orders):

(12) $$\begin{array}{l} \mathrm{cov}[X_{i}(A_{i}),X_{j}(A_{j})]\\ \;=\lambda \sum_{D^{\prime }⊃\{i,j\}}p_{D^{\prime }}\int P\left(t+Y_{i}\in A_{i},t+Y_{j}\in A_{j}\;|\;Y\sim Q_{D^{\prime }}\right)dt. \end{array}$$

In other words, the covariance of the counting processes is given by the weighted sum of the probabilities that the (*i, j*) marginal of the shift distributions yield values in the appropriate sets. The weights are the intensities of each corresponding component processes ξ(*D*) which contribute events to both of the processes *i* and *j*.

In the case that *Q*_*D*_ ≡ *Q*, Equation (12) reduces to the solution given in Bäuerle and Grübel (2005). Using the tail probabilities defined in Equation (8), if *Q*_*D*_ ≡ *Q* for all *D*, the integral in Equation (12) no longer depends on the subset *D*′, and the equation may be written as

$$\begin{array}{l} \mathrm{cov}[X_{i}(A_{i}),X_{j}(A_{j})]\\ \;=\lambda {\bar{p}}_{\left\{i,j\right\}}\int P\left(t+Y_{i}\in A_{i},t+Y_{j}\in \;A_{j}\;|\;Y\sim Q\right)dt. \end{array}$$

Using Equation (12), we may also compute the second cross-cumulant density (also called the *covariance density*) of the processes. From the definition of the cross-cumulant density [Equation (24) in the Methods], this is given by

(13) $$\begin{array}{l} {\text{κ}}_{ij}^{X}(\tau )=\underset{\Delta t\to 0}{\lim}\frac{\mathrm{cov}[X_{i}([0,\Delta t)),X_{j}([\tau ,\tau +\Delta t))]}{\Delta t^{2}}\\ \;\;\;\;\;\;\;\;\;=\lambda \sum_{D^{\prime }⊃\{i,j\}}p_{D^{\prime }}\\ \;\;\;\;\;\;\;\;\;\int^{\text{​}}\text{​​}\underset{\Delta t\to 0}{\lim}\text{​​}\frac{P(t+Y_{i}\in [0,\Delta t),t+Y_{j}\in [\tau ,\tau +\Delta t)\;|\;Y\sim Q_{D^{\prime }})}{\Delta t^{2}}dt. \end{array}$$

Before continuing, we note that given a random vector **Y** = (*Y*_1_, …, *Y*_*N*_) ~ *Q*, where *Q* has density *q*(*y*_1_, …, *y*_*N*_), the vector **Z** = (*Y*_2_− *Y*_1_, …, *Y*_*N*_− *Y*_1_) has density *q*_*Z*_ given by

(14) $$q_{Z}(\tau_{1},\ldots ,\tau_{N-1})=\int q(t,t+\tau_{1},\ldots ,t+\tau_{N-1})dt.$$

Assuming that the distributions *Q*_*D*′_ have densities *q*_*D*′_, and denoting by *q*^{*i, j*}^_*D*′_ the bivariate marginal density of the variables *Y*_*i*_, *Y*_*j*_ under *Q*_*D*′_, we have that

(15) $$\kappa_{ij}^{X}(\tau )=\lambda \sum_{D^{\prime }⊃\{i,j\}}p_{D^{\prime }}\int q_{D^{\prime }}^{\left\{i,j\right\}}(t,t+\tau )dt.$$

According to Equation (14), the integrals present in Equation (15) are simply the densities of the variables *Y*_*j*_− *Y*_*i*_, where **Y** ~ *Q*_*D*′_.

Thus κ^**X**^_*ij*_(τ), which captures the additional probability for events in the marginal processes *X*_*i*_ and *X*_*j*_ separated by τ units of time beyond what can be predicted from lower order statistics is given by a weighted sum (in this case, the lower order statistics are marginal intensities—see the discussion around Equation (24) of the Methods). The weights are the “marking rates” λ*p*_*D*′_ for markings contributing events to both component processes, while the summands are the probabilities that the corresponding shift distributions yield a pair of shifts in the proper arrangement—specifically, the shift applied to the event as attributed to *X*_*i*_ precedes that applied to the event mapped to *X*_*j*_ by τ units of time. This interpretation of the cross-cumulant density is quite natural, and will carry over to higher order cross-cumulants of a GTaS process. However, as we show next, this extension is not trivial at higher cumulant orders.

#### 2.3.2. Third order cumulants

To determine the higher order cumulants for a GTaS process, one can again use the representation given in Equation (10). The distribution of a subset of three processes may be expressed in the form (see Figure 6B)

(16) $$\left(\begin{array}{c} X_{i}(A_{i})\\ X_{j}(A_{j})\\ X_{k}(A_{k}) \end{array}\right){=}_{\text{distr}}\left(\begin{array}{c} \zeta_{\left\{i,j,k\right\}}+\zeta_{\left\{i,j\right\}}+\zeta_{\left\{i,k\right\}}+\zeta_{\left\{i\right\}}\\ \zeta_{\left\{i,j,k\right\}}+\zeta_{\left\{i,j\right\}}+\zeta_{\left\{j,k\right\}}+\zeta_{\left\{j\right\}}\\ \zeta_{\left\{i,j,k\right\}}+\zeta_{\left\{i,k\right\}}+\zeta_{\left\{j,k\right\}}+\zeta_{\left\{k\right\}}, \end{array}\right),$$

where, for simplicity, we suppressed the arguments of the different ζ_*D*_ on the right hand side. Again, the processes in the representation are independent and Poisson distributed. The variable ζ_{*i, j, k*}_ is the sum of all random variables ξ(*D*) (see Equation 9) with *D* ⊃ {*i, j, k*}, while the variable ζ_{*i, j*}_ is now the sum of all ξ(*D*) with *D* ⊃ {*i, j*}, but *k* ∉ *D*. The rest of the variables are defined likewise. Using properties (C1) and (C2) of cumulants given in the Methods, and assuming that *i, j, k* are distinct indices, we have

$$\kappa (X_{i}(A_{i}),X_{j}(A_{j}),X_{k}(A_{k}))=\kappa_{3}(\zeta_{\left\{i,j,k\right\}})=E[\zeta_{\left\{i,j,k\right\}}].$$

The second equality follows from the fact that all cumulants of a Poisson distributed random variable equal its mean. Similar to Equation (12), we may write

$$\kappa (X_{i}(A_{i}),X_{j}(A_{j}),X_{k}(A_{k}))\text{​}=\text{​}\lambda \sum_{D^{\prime }⊃\{i,j,k\}}p_{D^{\prime }}\int P(t+Y_{i}\in A_{i},t+\;Y_{j}\in A_{j},t+Y_{k}\in A_{k}|Y\sim Q_{D^{\prime }})\text{​}dt.$$

The third cross-cumulant density is then given similarly to the second order function by

$$\kappa_{ijk}^{X}(\tau_{1},\tau_{2})=\lambda \sum_{D^{\prime }⊃\{i,j,k\}}p_{D^{\prime }}\int q_{D^{\prime }}^{\left\{i,j,k\right\}}(t,t+\tau_{1},t+\tau_{2})dt.$$

Here, we have again assumed the existence of densities *q*_*D*′_, and denoted by *q*^{*i, j, k*}^_*D*′_ the joint marginal density of the variables *Y*_*i*_, *Y*_*j*_, *Y*_*k*_ under *q*_*D*′_. The integrals appearing in the expression for the third order cross-cumulant density are the probability densities of the vectors (*Y*_*j*_− *Y*_*i*_, *Y*_*k*_− *Y*_*i*_), where **Y** ~ *Q*_*D*′_.

#### 2.3.3. General cumulants

Finally, consider a general subset of *k* distinct members of the vector counting process **X** as in Equation (10). The following theorem provides expressions for the cross-cumulants of the counting processes, as well as the cross-cumulant densities, in terms of model parameters in this general case. The proof of Theorem 1.1 is given in the Appendix.

**Theorem 1.1.** *Let* **X** *be a joint counting process of GTaS type with total intensity λ, marking distribution (p_D_)_D ⊂ 𝔻_, and family of shift distributions (Q_D_)_D ⊂ 𝔻_. Let A_1_, …, A_k_ be arbitrary sets in* 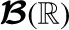
*, and $\bar{D}=\{i_{1},\ldots ,i_{k}\}\subset 𝔻$ with $|\bar{D}|=k$. The cross-cumulant of the counting processes may be written*

(17) $$\begin{array}{l} \kappa (X_{i_{1}}(A_{1}),\ldots ,X_{i_{k}}(A_{k}))\\ \;=\lambda \sum_{D^{\prime }⊃\bar{D}}p_{D^{\prime }}\int P(t1+Y^{\bar{D}}\in A_{1}\times \cdots \times \;A_{k}|Y\sim Q_{D^{\prime }})dt \end{array}$$

*where $Y^{\bar{D}}$ represents the projection of the random vector **Y** onto the dimensions indicated by the members of the set $\bar{D}$. Furthermore, assuming that the shift distributions possess densities (q_D_)_D ⊂ 𝔻_, the cross-cumulant density is given by*

(18) $$\begin{array}{l} \kappa_{i_{1}\cdots i_{k}}^{X}(\tau_{1},\ldots ,\tau_{k-1})\\ \;=\lambda \sum_{D^{\prime }⊃\bar{D}}p_{D^{\prime }}\int q_{D^{\prime }}^{\bar{D}}(t,t+\tau_{1},\cdots ,t+\tau_{k-1})dt, \end{array}$$

*where $q_{D^{\prime }}^{\bar{D}}$ indicates the kth order joint marginal density of q_D′_ in the dimensions of $\bar{D}$.*

An immediate corollary of Theorem 1.1 is a simple expression for the infinite-time-window cumulants, obtained by integrating the cumulant density across all time lags τ_*i*_. From Equation (A8), we have

(19) $$\begin{array}{l} \gamma_{i_{1}\cdots i_{k}}^{X}(\infty )=\int \cdots \int \kappa_{i_{1}\cdots i_{k}}^{X}(\tau_{1},\ldots ,\tau_{k-1})d\tau_{k-1}\cdots d\tau_{1}\\ \;=\lambda \sum_{D^{\prime }⊃\bar{D}}p_{D^{\prime }}\cdot 1=\lambda {\bar{p}}_{\bar{D}}. \end{array}$$

This shows that the infinite time window cumulants for a GTaS process are non-increasing with respect to the ordering of sets, i.e.,

$$\gamma_{i_{1}\cdots i_{k}}^{X}(\infty )\geq \gamma_{i_{1}\cdots i_{k}i_{k+1}}^{X}(\infty ).$$

We conclude this section with a short technical remark: Until this point, we have considered only the cumulant structure of sets of *unique* processes. However occasionally, one may wish to calculate a cumulant for a set of processes including repeats. Take, for example, a cumulant κ(*X*_1_(*A*_1_), *X*_1_(*A*_2_), *X*_3_(*A*_3_)). Owing to the marginally Poisson nature of the GTaS process, we would have (referring to the Methods for cumulant definitions)

(20) $$\begin{array}{l} \kappa (X_{1}(A_{1}),X_{1}(A_{2}),X_{3}(A_{3}))\\ \;=\kappa_{\left(2,1\right)}(X_{1}(A_{1}\cap A_{2}),X_{3}(A_{3}))\text{ if }X\sim \text{ GTaS}. \end{array}$$

For a general counting process **X**, it may be shown that

(21) $$\kappa_{113}^{X}(\tau_{1},\tau_{2})=\delta (\tau_{1})\kappa_{13}^{X}(\tau_{2})+\;“\text{non-singular contributions}”.$$

In addition, the second order auto-cumulant density may be written (Cox and Isham, 1980)

$$\kappa_{ii}^{X}(\tau )=r_{i}\delta (\tau )+\;“\text{non-singular contributions}”,$$

where *r*_*i*_ is the stationary rate. The singular contribution shown in Equation (21) at third order is in analogy to the delta contribution proportional to the firing rate which appears in the second-order auto-cumulant density. For a GTaS process, the non-singular contributions in Equation (21) are identically zero, following directly from Equation (20). Expressions similar to Equations (20, 21) hold for general cases.

## 3. Discussion

We have introduced a general method of generating spike trains with flexible spatiotemporal structure. The GTaS model is completely analytically tractable: all statistics of interest can be obtained directly from the distributions used to define it. It is based on an intuitive method of selecting and shifting point processes from a “mother” train. Moreover, the GTaS model can be used to easily generate partially synchronous states, cluster firing, cascading chains, and other spatiotemporal patterns of neural activity.

Processes generated by the GTaS model are naturally described by cumulant densities of pairwise and higher orders. This raises the question of whether such statistics are readily computable from data, so that realistic classes of GTaS models can be defined in the first place. One approach is to fit mechanistic models to data, and to use the higher order structure that is generated by the underlying mechanisms (Yu et al., 2011). A synergistic blend of other methods with the GTaS framework may also be fruitful—for example, the CuBIC framework of Staude et al. (2010) could be used to determine relevant marking orders, and the parametrically-described GTaS process could then be fit to allow generation of surrogate data after selection of appropriate classes of shift distributions. When it is necessary to infer higher order structure in the face of data limitations, population cumulants are an option to increase statistical power (albeit at the cost of spatial resolution; see Figure 4).

While the GTaS model has flexible higher order structure, it is always marginally Poisson. While throughout the cortex spiking is significantly irregular (Holt et al., 1996; Shadlen and Newsome, 1998), the level of variability differs across cells, with Fano factors ranging from below 0.5 to above 1.5—in comparison with the Poisson value of 1 (Churchland et al., 2010). Changes in variability may reflect cortical states and computation (Litwin-Kumar and Doiron, 2012; White et al., 2012). A model that would allow flexible marginal variability would therefore be very useful. Unfortunately, the tractability of the GTaS model is closely related to the fact that the marginal processes are Poisson. Therefore, an immediate generalization does not seem possible.

A number of other models have been used to describe population activity. Maximum entropy (ME) approaches also result in models with varied spatial activity; these are defined based on moments or other averaged features of multivariate spiking activity (Schneidman et al., 2006; Roudi et al., 2009). Such models are often used to fit purely spatial patterns of activity, though (Tang et al, 2008; Marre et al., 2009) have extended the techniques to treat temporal correlations as well. Generalized linear models (GLMs) have been used successfully to describe spatiotemporal patterns at second (Pillow et al., 2008), and third order (Ohiorhenuan et al., 2010). In comparison to the present GTaS method, both GLMs and ME models are more flexible. They feature well-defined approaches for fitting to data, including likelihood-based methods with well-behaved convexity properties. What the GTaS method contributes is an explicit way to generate population activity with explicitly specified high order spatio-temporal structure. Moreover, the lower order cumulant structure of a GTaS process can be modified independently of the higher order structure, though the reverse is not true.

There are a number of possible implications of such spatio-temporal structure for communication within neural networks. In section 2.2.3, we showed that these temporal correlations can play a role similar to that of spatial correlations established in Kuhn et al. (2003) for determining network input-output transfer. Our model allowed us to examine that impact of such temporal correlations on the network-level gain of a downstream population (cascade amplification factor). Even in a very simple network it was clear that the strength of the response is determined jointly by the temporal structure of the input to the network, and the connectivity within the network. Kuhn et al. examined the effect of higher order structure on the firing rate gain of an integrate-and-fire neuron by driving it with a mixture of SIP or MIP processes (Kuhn et al., 2003). However, in these studies, only the spatial structure of higher order activity was varied. The GTaS model allows us to concurrently change the temporal structure of correlations. In addition, the precise control of the cumulants allows us to derive models which are equivalent up to a certain cross-cumulant order, when the configuration of marking probabilities and shift distributions allow it (as for the SIP and MIP processes of Kuhn et al. (2003), which are equivalent at second order).

Such patterns of activity may be useful when experimentally probing dendritic information processing (Gasparini and Magee, 2006), synaptic plasticity (Pfister and Gerstner, 2006; Gjorgjieva et al., 2011), or investigating the response of neuronal networks to complex patterns of input (Kahn et al., 2013). Spatiotemporal patterns may also be generated by cell assemblies (Bathellier et al., 2012). The firing in such assemblies can be spatially structured, and this structure may not be reflected in the activity of participating cells. Assemblies can exhibit persistent patterns of firing, sometimes with millisecond precision (Harris et al., 2002). The GTaS framework is well suited to describe exactly such activity patterns. The examples we presented can be easily extended to generate more complex patterns of activity with overlapping cell assemblies, different cells leading the activity, and other variations.

Understanding impact of spatiotemporal patterns on neural computations remains an open and exciting problem. Progress will require coordination of computational, theoretical, and experimental work—the latter taking advantage of novel stimulation techniques. We hope that the GTaS model, as a practical and flexible method for generating high-dimensional, correlated spike trains, will play a significant role along the way.

## 4. Methods

### 4.1. Cumulants as a measure of dependence

We first define *cross-cumulants* (also called *joint cumulants*) (Stratonovich and Silverman, 1967; Kendall et al., 1969; Gardiner, 2009) and review some important properties of these quantities. Define the cumulant generating function *g* of a random vector **X** = (*X*_1_, …, *X*_*N*_) by

$$g(t_{1},\ldots ,t_{N})=\log\left(E[\exp\left(\sum_{j=1}^{N}t_{j}X_{j}\right)]\right).$$

The **r**-cross-cumulant of the vector **X** is given by

$$\kappa_{r}(X)=\frac{\partial^{\left|r\right|}}{\partial t_{1}^{r_{1}}\cdots \partial t_{N}^{r_{N}}}g(t_{1},\ldots ,t_{N}){|}_{t_{1}=\cdots =t_{N}=0}.$$

where **r** = (*r*_1_, …, *r*_*N*_) is a *N*-vector of positive integers, and |**r**| = ∑^*N*^_*i* = 1_ *r*_*i*_. We will generally deal with cumulants where all variables are considered at first order, without excluding the possibility that some variables are duplicated. In this case, we define the cross-cumulant κ(**X**), of the variables in the random vector **X** = (*X*_1_, …, *X*_*N*_) as

$$\begin{array}{l} \;\kappa (X):=\kappa_{1}(X)=\frac{\partial^{N}}{\partial t_{1}\cdots \partial t_{N}}g(t_{1},\ldots ,t_{N}){|}_{t_{1}=\cdots =t_{N}=0}\\ \text{where }1=(1,\ldots ,1). \end{array}$$

This relationship may be expressed in combinatorial form:

(22) $$\kappa (X_{1},\ldots ,X_{N})={\sum_{\pi }\left(|\pi |-1)!(-1\right)}^{|\pi |-1}\prod_{B\in \pi }E\left[\prod_{i\in B}X_{i}\right]$$

where π runs through all partitions of 𝔻 = {1, …, *N*}, and *B* runs over all blocks in a partition π. More generally, the **r**-cross-cumulant may be expressed in terms of moments by expanding the cumulant generating function as a Taylor series, noting that

$$\begin{array}{l} g(t_{1},\ldots ,t_{N})=\sum_{r}\frac{\kappa_{r}(X_{1},\ldots ,X_{N})}{r!}x_{1}^{r_{1}}\cdots x_{d}^{r_{N}}\text{ with}\\ \;r!=\prod_{i=1}^{N}r_{i}!, \end{array}$$

similarly expanding the moment generating function *M*(*t*) = *e*^*g*(*t*)^, and matching the polynomial coefficients. Note that the *n*th cumulant κ_*n*_ of a random variable *X* may be expressed as a joint cumulant via

$$\kappa_{n}(X)=\kappa (\underset{\text{n copies of }X}{\underset{︸}{X,\ldots ,X)}}.$$

We will utilize the following two principal properties of cumulants (Brillinger, 1965; Stratonovich and Silverman, 1967; Mendel, 1991; Staude et al., 2010):

(C1) Multilinearity - for any random variables *X, Y*, {*Z*_*i*_}^*N*^_*i* = 2_, we have
  $$\kappa (aX+bY,Z_{2},\ldots ,Z_{N})=a\kappa (X,Z_{2},\ldots ,Z_{N})+\;b\kappa (Y,Z_{2},\ldots ,Z_{N}).$$
  This holds regardless of dependencies amongst the random variables.
(C2) If any subset of the random variables in the cumulant argument is independent from the remaining, the cross-cumulant is zero—i.e., if {*X*_1_, …, *X*_*N*_1__} and {*Y*_1_, …, *Y*_*N*_2__} are sets of random variables such that each *X*_*i*_ is independent from each *Y*_*j*_, then
  $$\kappa_{\left(r_{X},r_{Y}\right)}(X_{1},\ldots ,X_{N_{1}},Y_{1},\ldots ,Y_{N_{2}})=0\text{ for all }r_{X}\in {\mathbb{N}}_{+}^{N_{1}},r_{Y}\in {\mathbb{N}}_{+}^{N_{2}}.$$

To exhibit another key property of cumulants, consider a 4-vector **X** = (*X*_1_, *X*_2_, *X*_3_, *X*_4_) with non-zero fourth cumulant and a random variable *Z* independent of each *X*_*i*_. Define **Y** = (*X*_1_ + *Z, X*_2_ + *Z, X*_3_ + *Z, X*_4_). Using properties (C1), (C2) above, it follows that

$$\kappa (Y_{1},Y_{2},Y_{3})=\kappa (X_{1},X_{2},X_{3})+\kappa_{3}(Z).$$

On the other hand, it is also true that

κ(Y)=κ(X),

that is, adding the variable *Z* to only a subset of the variables in **X** results in changes to cumulants involving only that subset, but *not* to the joint cumulant of the entire vector. In this sense, an *r*th order cross-cumulant of a collection of random variables captures exclusively dependencies amongst the collection which cannot be described by cumulants of lower order. In the example above, only the joint statistical properties of a subset of **X** were changed. As a result, the total cumulant κ(**X**) remained fixed.

From Equation (22), it is apparent that κ(*X*_*i*_) = **E**[*X*_*i*_], and κ(*X*_*i*_, *X*_*j*_) = **cov**[*X*_*i*_, *X*_*j*_]. In addition, the third cumulant, like the second, is equal to the corresponding central moment:

$$\kappa (X_{i},X_{j},X_{k})=E[(X_{i}-E[X_{i}])(X_{j}-E[X_{j}])(X_{k}-E[X_{k}])].$$

As cumulants and central moments agree up to third order, central moments up to third order inherit the properties discussed above at these orders. On the other hand, the fourth cumulant is *not* equal to the fourth central moment. Rather:

(23) $$\begin{array}{l} \kappa (X_{i},X_{j},X_{k},X_{l})\\ \;=E[(X_{i}-E[X_{i}\text{]})(X_{j}-E[X_{j}])(X_{k}-E[X_{k}])(X_{l}-E[X_{l}])]\\ -\mathrm{cov}[X_{i},X_{j}]\mathrm{cov}[X_{k},X_{l}]-\mathrm{cov}[X_{i},X_{k}]\mathrm{cov}[X_{j},X_{l}]\\ \;-\mathrm{cov}[X_{i},X_{l}]\mathrm{cov}[X_{j},X_{k}]. \end{array}$$

Higher cumulants have similar (but more complicated) expansions in terms of central moments. Accordingly, central moments of fourth and higher order do not inherit properties (C1), (C2).

### 4.2. Temporal statistics of point processes

In the Results, we present an extension of previous work (Bäuerle and Grübel, 2005) in which we construct and analyze multivariate counting processes **X** = (*X*_1_, …, *X*_*N*_) where each *X*_*i*_ is marginally Poisson.

Formally, a counting process **X** is an integer-valued random measure on 
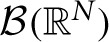
. Evaluated on subset *A*_1_ × … × *A*_*N*_ of 
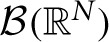
, the random vector (*X*_1_(*A*_1_), …, *X*_*N*_(*A*_*N*_)) counts events in *d* distinct categories whose times of occurrence fall in to the sets *A*_*i*_. A good general reference on the properties of counting processes (marginally Poisson and otherwise) is Daley and Vere-Jones (2002).

The assumption of Poisson marginals implies that for a set 
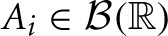
, the random variable *X*_*i*_(*A*_*i*_) follows a Poisson distribution with mean λ_*i*_ ℓ(*A*_*i*_), where ℓ is the Lebesgue measure on ℝ, and λ_*i*_ is the (constant) rate for the *i*th process. The processes under consideration will further satisfy a joint stationarity condition, namely that the distribution of the vector (*X*_1_(*A*_1_ + *t*), …, *X*_*N*_(*A*_*N*_ + *t*)) does not depend on *t*, where *A*_*i*_ + *t* denotes the translated set {*a* + *t* : *a* ∈ *A*_*i*_}.

We now consider some common measures of temporal dependence for jointly stationary vector counting processes. We will refer to the quantity *X*_*i*_[0, *T*] as the *spike count* of process *i* over [0, *T*]. The quantity γ^**X**^_*i*_1_… *i*_*k*__(*T*) (which we will refer to as a *spike count cumulant*) is given by

$$\gamma_{i_{1}\cdots i_{k}}^{X}(T)=\frac{1}{T}\kappa [X_{i_{1}}[0,T],\ldots ,X_{i_{k}}[0,T]]$$

measures *k*th order correlations amongst spike counts for the listed processes which occur over windows of length *T*. At second order, γ^**X**^_*ij*_(*T*) measures the covariance of the spike counts of processes *i, j* over a common window of length *T*. The infinite window spike count cumulant quantifies dependencies in the spike counts of point processes over arbitrarily long windows, and is given by

$$\gamma_{i_{1}\cdots i_{k}}^{X}(\infty )=\underset{T\to \infty }{\lim}\gamma_{i_{1}\cdots i_{k}}^{X}(T).$$

A related measure is the *k*th order cross-cumulant density κ^*X*^_*i*_1_, …, *i*_*k*__(τ_1_, …, τ_*k* − 1_), defined by

(24) $$\begin{array}{l} \kappa_{i_{1}\cdots i_{k}}^{X}(\tau_{1},\ldots ,\tau_{k-1})=\underset{\Delta t\to 0}{\lim}\frac{1}{\Delta t^{k}}\kappa [X_{i_{1}}[0,\Delta t],\\ \;X_{i_{2}}[\tau_{1},\tau_{1}+\;\Delta t],\ldots ,X_{i_{k}}[\tau_{k-1},\tau_{k-1}+\Delta t]]. \end{array}$$

The cross-cumulant density should be interpreted as a measure of the likelihood—above what may be expected from knowledge of the lower order cumulant structure—of seeing events in processes *i*_2_, …, *i*_*k*_ at times τ_1_ + *t*, …, τ_*k* − 1_ + *t*, conditioned on event in process *i*_1_ at time *t*. The infinite window spike count cumulant is equal to the total integral under the cross-cumulant density,

$$\gamma_{i_{1}\cdots i_{k}}^{X}(\infty )=\int \cdots \int \kappa_{i_{1}\cdots i_{k}}^{X}(\tau_{1},\ldots ,\tau_{k-1})d\tau_{k-1}\cdots d\tau_{1}.$$

As an example, we again consider the familiar second-order cross-cumulant density κ^*X*^_*ij*_(τ)—often referred to as the *cross-covariance density* or *cross-correlation function*. Defining the conditional intensity *h*_*ij*_(τ) of process *j*, conditioned on process *i* to be

$$h_{ij}^{X}(\tau )=\underset{\Delta t\to 0}{\lim}\frac{1}{\Delta t}P(X_{j}[\tau ,\tau +\Delta t]>0|X_{i}[0,\Delta t]>0),$$

that is, the intensity of *j* conditioned on an event in process *i* which occurred τ units of time in the past, then it is not difficult to show that

$$\kappa_{ij}^{X}(\tau )=\lambda_{i}h_{ij}(\tau )-\lambda_{i}\lambda_{j}.$$

That is, the second order cross-cumulant density supplies the probability of chance of observing an event attributed to process *i*, followed by one attributed to process *j*, τ units of time later, above what would be expected from knowledge of first order statistics (given by the product of the marginal intensities, λ_*i*_ λ_*j*_). More generally, at higher orders, the cross-cumulant density should be interpreted as a measure of the likelihood (above what may be expected from knowledge of the lower order correlation structure) of seeing events attribute to processes *i*_2_, …, *i*_*k*_ at times τ_1_ + *t*, …, τ_*k* − 1_ + *t*, conditioned on an event in process *i*_1_ at time *t*.

Another statistic useful in the study of a correlated vector counting process **X** is the *population cumulant density*. At second-order, the population cumulant density for *X*_*i*_ takes the form (Luczak et al., 2013)

$$\kappa_{i,\text{pop}}^{X}(\tau )=\sum_{j\neq i}\kappa_{ij}^{X}(\tau ).$$

More generally, the *k*th order population cumulant density corresponding to the processes *X*_*i*_1__, …, *X*_*i*_*k* − 1__ is given by

(25) $$\kappa_{i_{1}\cdots i_{k-1},\text{pop}}^{X}(\tau_{1},\ldots ,\tau_{k-1})=\sum_{j\neq i_{1},\ldots ,i_{k}}\kappa_{i_{1}\cdots i_{k-1}j}^{X}(\tau_{1},\ldots ,\tau_{k-1}).$$

## Funding

This work was supported by NSF grants DMS-0817649, DMS-1122094, a Texas ARP/ATP award to Krešimir Josić, and by a Career Award at the Scientific Interface from the Burroughs Wellcome Fund and NSF Grant DMS-1122106 to Eric Shea-Brown.

### Conflict of interest statement

The authors declare that the research was conducted in the absence of any commercial or financial relationships that could be construed as a potential conflict of interest.

## Appendix

### Proof of the distributional representation of the GTaS model in equation (9)

The construction of the GTaS model allows us to provide a useful distributional representation of the process. We describe this representation in a theorem that generalizes Theorem 1 in Bäuerle and Grübel (2005). This theorem also immediately implies that the GTaS process is marginally Poisson.

Some definitions are required: first, for subsets  and *D, D*′ ⊂ 𝔻 with *D* ⊂ *D*′, let

$$\begin{array}{l} M(D,D^{\prime };A_{1},\ldots ,A_{N}):=B_{1}\times \cdots \times B_{N}\text{ with }B_{i}\\ \;:=\{\begin{array}{ll} A_{i}, & \text{for }i\in D,\\ A_{i}^{c}, & \text{for }i\in D^{\prime }\backslash D,\\ \mathbb{R}, & \text{otherwise} \end{array} \end{array}$$

In addition, setting **1** = (1, …, 1) to be the *N*-dimensional vector with all components equal to unity, and if *Q*_*D*_ is a measure on ℝ^*N*^, then we define the measure ν(*Q*_*D*_) by

The measure ν(*Q*_*D*_) can be interpreted as giving the *expected* Lebesgue measure of the subset *L* of ℝ for which uniform shifts by the elements of *L* translate a random vector **Y** ~ *Q*_*D*_ in to *A*. Heuristically, one may imagine sliding the vector **Y** over the whole real line, and counting the number of times every coordinate ends up in the “right” set—the projection of *A* onto that dimension. In equation form, this means

(A2) $$\nu (Q_{D})(A)=E_{Y}[\ell (\{t\in \mathbb{R}:Y+t1\in A\})|Y\sim Q_{D}].$$

where the subscript **Y** indicates that we take the average over the distribution of **Y** ~ *Q*_*D*_. A short proof of this representation is presented below. We now present the theorem, with a proof indicating adjustments necessary to that of Bäuerle and Grübel (2005).

**Theorem 0** *Let X be an N-dimensional counting process of GTaS type with base rate λ, thinning mechanism p = (p_D_)_D ⊂ 𝔻_, and family of shift distributions (Q_D_)_D ⊂ 𝔻_. Then, for any Borel subsets A_1_, …, A_N_ of the real line, we have the following distributional representation:*

(A3) $$\left(\begin{array}{c} X_{1}(A_{1})\\ \vdots \\ X_{N}(A_{N}) \end{array}\right){=}_{\text{distr}}\left(\begin{array}{c} \sum_{D\;∋\;1}\xi (D;A_{1},\ldots ,A_{N})\\ \vdots \\ \sum_{D\;∋\;d}\xi (D;A_{1},\ldots ,A_{N}) \end{array}\right),$$

*where the random variables ξ(D; A_1_, …, A_N_), ∅ ≠ D ⊂ 𝔻, are independent and Poisson distributed with*

$$E[\xi (D;A_{1},\ldots ,A_{N})]=\lambda \sum_{D^{\prime }⊃D}p_{D^{\prime }}\nu (Q_{D^{\prime }})(M(D,D^{\prime };A_{1},\ldots ,A_{N})).$$

*Proof*. For each marking *D*′ ⊂ 𝔻, define **X**^*D*′^ to be an independent TaS (Bäuerle and Grübel, 2005) counting process with mother process rate λ*p*_*D*′_, shift distribution *Q*_*D*′_, and markings (*p*^*D*′^_*D*_)_*D* ⊂ 𝔻_ where *p*^*D*′^_*D*_ = 1 if *D* = *D*′ and is zero otherwise (i.e., the only possible marking for **X**^*D*′^ is *D*′). We first claim that

(A4) $$X{=}_{\text{distr}}\sum_{D^{\prime }}X^{D^{\prime }}.$$

To see this, note that spikes in the mother process of the GTaS process of **X** marked for a set *D*′ occur at a rate λ*p*_*D*′_, which is the rate of the process **X**^*D*′^. In addition, these event times are then shifted by *Q*_*D*′_, exactly as they are for **X**^*D*′^. Thus, the distribution of event times (and hence the counting process distributions) are equivalent.

Let *A*_1_, …, *A*_*N*_ be any Borel subsets of the real line. Applying Theorem 1 of Bäuerle and Grübel (2005) to each **X**^*D*′^ gives the following distributional representation:

(A5) $$\left(\begin{array}{c} X_{1}^{D^{\prime }}(A_{1})\\ \vdots \\ X_{N}^{D^{\prime }}(A_{N}) \end{array}\right){=}_{\text{distr}}\left(\begin{array}{c} \sum_{D∋1}\xi^{D^{\prime }}(D;A_{1},\ldots ,A_{N})\\ \vdots \\ \sum_{D∋N}\xi^{D^{\prime }}(D;A_{1},\ldots ,A_{N}) \end{array}\right),$$

where the random variables ξ^*D*′^(*D*;, *A*_1_, …, *A*_*N*_) are taken to be identically zero unless *D* ⊂ *D*′. In the latter case, they are independent and Poisson distributed with

$$\begin{array}{l} E[\xi^{D^{\prime }}(D;A_{1},\ldots ,A_{N})]\\ \;=\lambda p_{D^{\prime }}\sum_{D^{\prime \prime }⊃D}p_{D^{\prime \prime }}^{D^{\prime }}\nu (Q_{D^{\prime }})(M(D,D^{\prime \prime };A_{1},\ldots ,A_{N}))\\ \;=\lambda p_{D^{\prime }}\nu (Q_{D^{\prime }})(M(D,D^{\prime };A_{1},\ldots ,A_{N})). \end{array}$$

The second equality above follows from the fact that *p*^*D*′^_*D*″_ = 1 if *D*″ = *D*′ and is zero otherwise.

Next, define

$$\begin{array}{l} \xi (D;A_{1},\ldots ,A_{N})=\sum_{D^{\prime }}\xi^{D^{\prime }}(D;A_{1},\ldots ,A_{N})\\ \;=\sum_{D^{\prime }⊃D}\xi^{D^{\prime }}(D;A_{1},\ldots ,A_{N}). \end{array}$$

As the sum of independent Poisson variables is again Poisson with rate equal to the sum of the rates, we have that ξ(*D*; *A*_1_, …, *A*_*N*_) is Poisson with mean

(A6) $$E[\xi (D;A_{1},\ldots ,A_{N})]=\lambda \sum_{D^{\prime }⊃D}p_{D^{\prime }}\nu (Q_{D^{\prime }})(M(D,D^{\prime };A_{1},\ldots ,A_{N})).$$

Finally, combining Equations (A4, A5), we may write

$$\begin{array}{l} \left(\begin{array}{c} X_{1}(A_{1})\\ \vdots \\ X_{N}(A_{N}) \end{array}\right){=}_{\text{distr}}\left(\begin{array}{c} \sum_{D^{\prime }}\sum_{D∋1}\xi^{D^{\prime }}(D;A_{1},\ldots ,A_{N})\\ \vdots \\ \sum_{D^{\prime }}\sum_{D∋N}\xi^{D^{\prime }}(D;A_{1},\ldots ,A_{N}) \end{array}\right),\\ \;=\left(\begin{array}{c} \sum_{D∋1}\sum_{D^{\prime }}\xi^{D^{\prime }}(D;A_{1},\ldots ,A_{N})\\ \vdots \\ \sum_{D∋N}\sum_{D^{\prime }}\xi^{D^{\prime }}(D;A_{1},\ldots ,A_{N}) \end{array}\right),\\ \;=\left(\begin{array}{c} \sum_{D∋1}\xi (D;A_{1},\ldots ,A_{N})\\ \vdots \\ \sum_{D∋N}\xi (D;A_{1},\ldots ,A_{N}) \end{array}\right), \end{array}$$

which, along with Equation (A6), establishes the theorem.

                                                                                □

A short note: The variable ξ(*D*; *A*_1_, …, *A*_*N*_) counts the number of points which are marked by a set *D*′ ⊃ *D*, but after shifting, only the points attributed to the processes with indices *i* ∈ *D* remain in the corresponding subsets *A*_*i*_. Thus, to determine the number of points attributed to the *i*th process which lie in *A*_*i*_ (*X*_*i*_(*A*_*i*_)), one simply sums the variables ξ for all *D* containing *i*, as in Equation (A3). Thus, the intensity of ξ(*D*; *A*_1_, …, *A*_*N*_),

$$\lambda p_{D^{\prime }}\nu (Q_{D^{\prime }})(M(D,D^{\prime };A_{1},\ldots ,A_{N})),$$

is simply the expected number of such points. Keeping in mind these natural interpretations of terms, Theorem 1 is easier to digest, and the result is not surprising.

### Proof of equation (27)

In Equation (A2), we gave a more intuitive representation of the measure ν(*Q*_*D*_) than the one first defined in Bäuerle and Grübel (2005), which we prove here. Suppose that *Q* is a measure on , and . Then we have

$$\begin{array}{l} \nu (Q)(A)=\int Q(A-t1)dt\\ \;=\iint 1_{A-t1}(y)Q(dy)dt\\ \;=\iint 1_{\left\{t\in \mathbb{R}:y+t1\in A\right\}}(t)dtQ(dy)\\ \;=\int \ell (\{t\in \mathbb{R}:y+t1\in A\})Q(dy)\\ \;=E_{Y}[\ell (\{t\in \mathbb{R}:Y+t\in A\})|Y\sim Q], \end{array}$$

thus proving Equation (A2)

### Proof of Theorem 1.1

**Theorem 1.1** *Let X be a joint counting process of GTaS type with total intensity λ, marking distribution (p_D_)_D ⊂ 𝔻_, and family of shift distributions (Q_D_)_D ⊂ 𝔻_. Let A_1_, …, A_k_ be arbitrary sets in* *, and $\bar{D}=\{i_{1},\ldots ,i_{k}\}\subset 𝔻$ with $|\bar{D}|=k$. The cross-cumulant of the counting processes may be written*

(A7) $$\kappa (X_{i_{1}}(A_{1}),\ldots ,X_{i_{k}}(A_{k}))=\lambda \sum_{D^{\prime }⊃\bar{D}}p_{D^{\prime }}\int P(t1+Y^{\bar{D}}\in A_{1}\times \cdots \times \;A_{k}|Y\sim Q_{D^{\prime }})dt$$

*where $Y^{\bar{D}}$ represents the projection of the random vector* **Y** *onto the dimensions indicated by the members of the set $\bar{D}$. Furthermore, assuming that the shift distributions possess densities (q_D_)_D ⊂ 2^𝔻^_, the cross-cumulant density is given by*

(A8) $$\begin{array}{l} \kappa_{i_{1}\cdots i_{k}}^{X}(\tau_{1},\ldots ,\tau_{k-1})\\ \;=\lambda \sum_{D^{\prime }⊃\bar{D}}p_{D^{\prime }}\int q_{D^{\prime }}^{\bar{D}}(t,t+\tau_{1},\cdots ,t+\tau_{k-1})dt, \end{array}$$

*where $q_{D^{\prime }}^{\bar{D}}$ indicates the kth order joint marginal density of q_D′_ in the dimensions of $\bar{D}$.*

*Proof*. First, as noted in the text, we may rewrite the distributional representation of Theorem 0 (Equation A3) as

(A9) $$\left(\begin{array}{c} X_{i_{1}}(A_{i_{1}})\\ \vdots \\ X_{i_{k}}(A_{i_{k}}) \end{array}\right){=}_{\text{distr}}\left(\begin{array}{c} \sum_{i_{1}\in D\subset \bar{D}}\zeta_{D}(A_{1},\ldots ,A_{N})\\ \vdots \\ \sum_{i_{k}\in D\subset \bar{D}}\zeta_{D}(A_{1},\ldots ,A_{N}) \end{array}\right)$$

where

(A10) $$\zeta_{D}(A_{1},\ldots ,A_{N})=\sum_{\begin{array}{l} \;D^{\prime }⊃D\\ (\bar{D}\backslash D)\cap D^{\prime }=\emptyset \end{array}}\xi (D^{\prime };A_{1},\ldots ,A_{N}).$$

Repeating the description from the main text, the processes ζ_*D*_ are comprised of a sum of all of the processes ξ(*D*′) (defined above, in Theorem 0) such that *D*′ contains all of the indices *D*, but no other indices which are part of the subset $\bar{D}$ under consideration. These sums are non-overlapping, implying that the ζ_*D*_ are also independent and Poisson.

Using the representation of Equation (A9), we first find that

$$\begin{array}{l} \kappa (X_{i_{1}}(A_{1}),\ldots ,X_{i_{k}}(A_{k}))=\kappa \left[\sum_{i_{1}\in D_{1}\subset \bar{D}}\zeta_{D_{1}}\;,\;\ldots \;,\;\sum_{i_{k}\in D_{k}\subset \bar{D}}\zeta_{D_{k}}\right]\\ \;=\sum_{i_{1}\in D_{1}\subset \bar{D}}\cdots \sum_{i_{k}\in D_{k}\subset \bar{D}}\kappa [\zeta_{D_{1}},\ldots ,\zeta_{D_{k}}]. \end{array}$$

where we suppressed the dependence of the variables ζ_*D*_ on the subsets *A*_*i*_. The first equality in the previous equation is simply the representation defined in Equation (A10), and the second is from the multilinear property of cumulants (property (C1) in the Methods). Note that the sums are over the sets *D*_1_, …, *D*_*k*_ satisfying the given conditions. Recall that, by construction, the Poisson processes ζ_*D*_ (see Equation A10) are independent for distinct marking sets. Accordingly, the cumulant κ[ζ_*D*_1__, …, ζ_*D*_*k*__] is zero unless *D*_1_ = … = *D*_*k*_, by property (C2) of cumulants—that is,

$$\begin{array}{l} \kappa [\zeta_{D_{1}}(A_{1},\ldots ,A_{N}),\ldots ,\zeta_{D_{k}}(A_{1},\ldots ,A_{N})]\\ \;=\{\begin{array}{ll} \kappa_{k}(\zeta_{\bar{D}}(A_{1},\ldots ,A_{N})) & D_{j}=\bar{D}\text{ for each }j\\ 0 & \text{otherwise} \end{array}. \end{array}$$

Hence,

(A11) $$\begin{array}{l} \kappa (X_{i_{1}}(A_{1}),\ldots ,X_{i_{k}}(A_{k}))=\kappa_{k}(\zeta_{\bar{D}}(A_{1},\ldots ,A_{N}))\\ \;=E[\zeta_{\bar{D}}(A_{1},\ldots ,A_{N})], \end{array}$$

where we have again used that all cumulants of a Poisson-distributed random variable are equal to its mean.

For what follows, taking *D*_0_, *D*′ ⊂ 𝔻 fixed with *D*_0_ ⊂ *D*′, the sets *M*(*D, D*′;*A*_1_, …, *A*_*N*_) with *D*_0_ ⊂ *D* ⊂ *D*′ are disjoint, and

(A12) $$\begin{array}{l} \cup_{D_{0}\subset D\subset D^{\prime }}M(D,D^{\prime };A_{1},\ldots ,A_{N})=B_{1}\times \cdots \times B_{N}\\ \text{ with }B_{i}=\{\begin{array}{ll} A_{i}, & i\in D_{0}\\ \mathbb{R}, & i\notin D_{0} \end{array}. \end{array}$$

In particular, note the independence of the above union from *D*′.

Substituting Equation (A10) in to (A11), we have

$$\begin{array}{l} \kappa (X_{i_{1}}(A_{1}),\ldots ,X_{i_{k}}(A_{k}))\\ \;=\sum_{D⊃\bar{D}}E[\xi (D;A_{1},\ldots ,A_{k}]\\ \;=\lambda \sum_{D⊃\bar{D}}\sum_{D^{\prime }⊃D}p_{D^{\prime }}\nu (Q_{D^{\prime }})(M(D,D^{\prime };A_{1},\ldots ,A_{N}))\\ \;=\lambda \sum_{D^{\prime }⊃\bar{D}}p_{D^{\prime }}\sum_{\bar{D}\subset D\subset D^{\prime }}\nu (Q_{D^{\prime }})(M(D,D^{\prime };A_{1},\ldots ,A_{N}))\\ \;=\lambda \sum_{D^{\prime }⊃\bar{D}}p_{D^{\prime }}\nu (Q_{D^{\prime }})(\cup_{\bar{D}\subset D\subset D^{\prime }}M(D,D^{\prime };A_{1},\ldots ,A_{N}))\\ \;=\lambda \sum_{D^{\prime }⊃\bar{D}}p_{D^{\prime }}\int P(t+Y^{\bar{D}}\in A_{1}\times \;\cdots \times A_{k}|Y\sim Q_{D^{\prime }})dt, \end{array}$$

where the third equality above is a simple exchange of the order of summation, and the fourth equality follows from the additivity of the measure ν(*Q*_*D*′_) over the disjoint sets *M*(*D, D*′;*A*_1_, …, *A*_*N*_). Finally, the fifth equality makes use of the independence of the set union on the fourth line from the set *D*′ as indicated by Equation (A12), the definition of the measure ν(*Q*_*D*′_) in Equation (A1) and the value of the set union given in Equation (A12).

This completes the proof of Equation (A7), and (A8) follows from the definition of the cross-cumulant density in Equation (24) of the Methods.

                                                                                □

### Other details

#### Parameters for figures in the text

*Figure 1*. For Figure 1, the GTaS process of size *N* = 6 consisted of only first order and population-level events which were assigned marking probabilities

$$p_{D}=\{\begin{array}{ll} 0.05 & D=𝔻\\ \frac{0.95}{6} & D=\{i\}\text{ for some }i\in 𝔻\\ 0 & \text{otherwise} \end{array}.$$

The rate of the mother process was λ = 0.5 kHz, and the shift times for population level events were generated as in section 2.2.2 with

$$T_{i}\sim \Gamma (2,1)-1,\;i=1,\ldots ,6,$$

where the Gamma distribution has density

$$f(t|k,\theta )=\frac{1}{\Gamma (k)\theta^{k}}x^{k-1}e^{-\frac{x}{\theta }}\Theta (t).$$

*Figures 3, 4*. For Figures 3, 4, the GTaS process of size *N* = 6 consisted of first and second order as well as population-level events. These events had marking probabilities

$$p_{D}=\{\begin{array}{ll} 0.05 & D=𝔻\\ \frac{0.95}{21} & D=\{i\},\{i,j\}\text{ for some }i,j\in 𝔻\\ 0 & \text{otherwise} \end{array}.$$

The rate of the mother process was λ = 0.5 kHz, and the shift times for population level events were generated as in section 2.2.2 with

$$T_{i}\sim \text{Exp}(0.5),\;i=1,\ldots ,6.$$

The shift times of the second order events were drawn from an independent Gaussian distribution with each coordinate having standard deviation 5 ms.

*Figure 5*. For Figure 5, the network parameters were *w*^in^ = 0.4, *w*^syn^ = 6, τ_syn_ = 0.1, τ_d_ = 1.75. The GTaS input had the same size as the network (*N* = 10). As in the example of Figures 3, 4, the GTaS input included first and second order as well as population level events. Here, we set

$$p_{D}=\{\begin{array}{ll} 0.2 & D=𝔻\\ \frac{0.95}{5} & D=\{i\},\{i,j\}\text{ for some }i,j\in 𝔻\\ 0 & \text{otherwise} \end{array}.$$

The rate of the mother process was λ = 1.5 kHz, and the shift times for population level events were generated as in section 2.2.2 with

$$T_{i}\sim \Gamma (k,\theta ),\;i=1,\ldots ,6.$$

The shift parameters *k*,θ (representing shape and scale) were determined by the given shift mean μ_shift_ and standard deviation σ_shift_ as

$$\mu_{\text{shift}}=k\theta ,\;\sigma_{\text{shift}}=\sqrt{k\theta^{2}}.$$

The shift times of the second order events were drawn from an independent Gaussian distribution with each coordinate having standard deviation 0.3 ms.
